# Symbiotic effectiveness and ecologically adaptive traits of native rhizobial symbionts of Bambara groundnut (*Vigna subterranea* L. Verdc.) in Africa and their relationship with phylogeny

**DOI:** 10.1038/s41598-019-48944-1

**Published:** 2019-09-02

**Authors:** Fadimata Y. I. Ibny, Sanjay K. Jaiswal, Mustapha Mohammed, Felix D. Dakora

**Affiliations:** 1 Department of Crop Sciences, Tshwane University of Technology, Private Bag X680, Pretoria, 0001 South Africa; 2 Chemistry Department, Tshwane University of Technology, Private Bag X680, Pretoria, 0001 South Africa

**Keywords:** Rhizobial symbiosis, Soil microbiology

## Abstract

Bambara groundnut (*Vigna subterranea* L. Verdc.) is an indigenous, drought-tolerant, underutilized African food legume, with the ability to fix atmospheric N_2_ in symbiosis with soil bacteria called rhizobia. The aim of this study was to assess the morpho-physiological, symbiotic and phylogenetic characteristics of rhizobia nodulating Bambara groundnut in Ghana, Mali and South Africa. The morpho-physiologically diverse isolates tested were also found to exhibit differences in functional efficiency and phylogenetic positions. Based on Enterobacterial Repetitive Intergenic Consensus (ERIC)-PCR banding patterns, the isolates were grouped into eight major clusters. The concentrations of Ca, Na and K in soils had a significant (p ≤ 0.01) effect on the distribution of rhizobia. Though many isolates were symbiotically very effective, the effectiveness index varied markedly (p ≤ 0.05) among them. Moreover, the isolates also exhibited tolerance to a wide range of NaCl (0.5–7%), streptomycin (50–500 µg.ml^−1^), and kanamycin (25–150 µg.ml^−1^) concentrations. Additionally, these isolates could produce 0.02 to 69.71 µg.ml^−1^ of indole-3-acetic acid (IAA) in tryptophan-supplemented medium, as well as solubilize tri-calcium phosphate. Phylogenetic analysis of these rhizobial isolates using 16S rRNA, *atpD*, *glnII*, *gyrB*, *recA* and symbiotic (*nifH* and *nodC*) gene sequences revealed distinct and novel evolutionary lineages related to the genus *Bradyrhizobium*, with some of them being very close to *Bradyrhizobium vignae*, *B*. *kavangense*, *B*. *subterraneum*, *B*. *elkanii* and *B*. *pachyrhizi*.

## Introduction

Bambara groundnut (*Vigna subterranea* L. Verdc.) belongs to the family Leguminosae (Fabaceae), sub family Faboidea^1^. It is a drought-tolerant underutilized African food legume commonly grown for its grain by subsistence farmers in sub-Saharan Africa^2^. The crop derived its name from a tribe called “Bambara” in Segou, central Mali. It is the third most important food legume in Africa after groundnut and cowpea, both in consumption and land area under cultivation^3^. The edible grain of this legume has high protein content (20.6%), carbohydrate (56.5%), fat (6.6%) and fibre (6.3%), which together make it a balanced meal^4^. The leaves are high in N and K, and therefore serve as an excellent protein-rich feed for animals. Its cultivation outside Africa includes India, Srilanka, Indonesia, the Philipines and Malayasia in Asia, as well as Brazil, Paraguay and Suriname in South America^5–7^. Bambara groundnut can produce high yields even in low nutrient soils, and where there is drought stress due to low soil moisture.

Bambara groundnut can fix about 4 to 200 kg N. ha^−1^ ^7^ through symbiotic relationship with soil bacteria called ‘rhizobia’^3^. There is little information on the biodiversity of rhizobia nodulating Bambara groundnut in African soils, except for a few studies which have shown that Bambara groundnut is nodulated by species of the genus *Bradyrhizobium*^3,8^. As rhizobial species nodulating most legumes can vary between geographical locations, it is important to continuously explore new geographic regions to identify rhizobia that are capable of effectively nodulating and promoting the growth of important but underutilized crop species such as Bambara groundnut. Biotic factors such as Rhizobiophages, antibiotic-producing microbes and edaphic factors have been shown to determine the distribution and population of resident soil rhizobia^9–13^. For instance, soil pH, P, temperature, salinity, antibiotics and moisture content have a strong effect on the diversity of rhizobia in diverse agro-ecological regions^14–21^. In some instances, indigenous rhizobia may develop strategies for survival under ecologically harsh conditions, in addition to occasionally exhibiting high levels of N_2_ fixation and plant growth promotion when compared to introduced commercial strains^12^.

Understanding the taxonomy and phylogenetic relationships among rhizobia is a first step towards improving legume productivity using biological N_2_ fixation^22^. The aim of this study was to study native rhizobia nodulating Bambara groundnut in soils of Ghana, Mali and South Africa, and to identify those that have ecologically-adapted traits for plant growth promotion and enhanced N_2_ fixation for use as inoculants.

## Results

A total of 201 bacterial isolates were isolated from root nodules of Bambara groundnut collected from farmers’ fields. The results showed that the soils used in this study were acidic in nature with high K concentration at Marapyane (246 mg.kg^−1^) in South Africa, and low levels in soil from Kamale (31 mg.kg^−1^) in Mali (Table 1).

**Table 1:** Geographic origin of isolates and the soil chemical properties of sampling locations.

Isolates	Origin	pH	Na	K	P	Ca
		mg.kg^−1^				Cmol.kg^−1^
TUTMa1 – TUTMa61	Marapyane, South Africa	5.2	35	246	8	4.76
TUTKa77 – TUTKa82	Kamale, Mali	5.7	8	31	6	2.59
TUTNa62 – TUTNa63	Narena, Mali	5.5	13	228	5	2.33
TUTNou64 – TUTNou76	Nougani, Mali	5.5	13	128	2	1.94
TUTGh83, 84, 87, 90	Googo, Ghana	4.6	16	140	15	2.67
TUTGh85,88, 89	Savelugu, Ghana	5.1	7	47	7	3.2

Out of the 201 isolates subjected to nodulation assay, 44% (89 isolates) were able to induce effective nodules on their respective Bambara groundnut host (landrace Uniswa red), while six isolates formed ineffective nodules. The remaining 106 isolates failed to nodulate the host plant. As expected, no nodules were found on roots of the uninoculated control plants, as well as the NO_3_^−^ fed seedlings.

### Morpho-physiological characterization of Bambara groundnut-nodulating bacterial isolates

**Figure 1:**
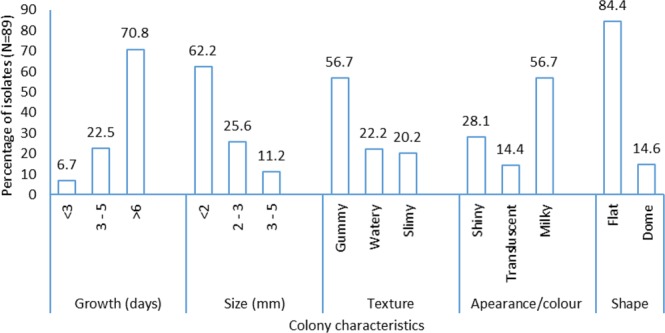
Morpho-physiological description of Bambara groundnut isolates (N = 89) from various locations in Ghana, Mali and South Africa.

The rhizobial isolates were phenotypically diverse in terms of colony appearance, shape, size (diameter), colour and opacity (Fig. 1). The growth periods of the colonies (number of days taken for colonies to appear on YMA) varied from 2 to 15 days. About 71% of the isolates were slow-growers and took 6 to 15 days to appear on yeast mannitol agar (YMA) plates, while the remaining isolates exhibited either intermediate or fast growth rates (Fig. 1). Moreover, 62% of the isolates revealed small colony sizes (<2 mm diameter), while a greater proportion were gummy in texture (57%), milkish in appearance (57%) and flat in shape (84%) (Fig. 1).

### Salinity tolerance

The rhizobial isolates differed in their response to sodium chloride concentrations. All the isolates tested grew in medium supplemented with 0.01% NaCl (control). However, an increase in NaCl concentration resulted in a reduction in the number of isolates that were tolerant to NaCl. Of the 89 nitrogen-fixing rhizobial isolates tested, 18 grew well on media containing up to 0.5% NaCl, one in up to 2% NaCl, 7 in up to 3% NaCl, one in up to 5% NaCl, while five isolates (TUTNou71, TUTMa36, TUTMa37, TUTNou68 and TUTGh90) could extended their tolerance level up to 7% NaCl (Table 2). A total of 56 isolates were susceptible to concentrations of NaCl above control (0.01%).

**Table 2:** Phenotypic traits of indigenous rhizobial symbionts of Bambara groundnut.

	NaCl	Antibiotic resistance		PSB halo-zone	IAA
		Streptomycin	Kanamycin		
	%	µg.mL^−1^		mm	µg.mL^−1^
TUTMa1	0.01	0	0	0	0
TUTMa2	0.01	500	0	0	1.50 ± 0.21
TUTMa3	0.5	100	0	0	0.034 ± 0.26
TUTMa4	0.01	0	0	0	0
TUTMa5	0.01	0	0	0	0
TUTMa6	0.5	500	0	0	0
TUTMa7	0.01	0	0	0	0
TUTMa8	0.5	500	0	0	0
TUTMa9	0.5	500	0	0	0
TUTMa10	0.01	0	0	0	0
TUTMa11	0.5	500	0	0	0
TUTMa12	0.5	0	0	0	0
TUTMa13	0.5	500	0	0	0
TUTMa14	0.5	500	0	0	0
TUTMa15	0.5	500	0	0	0
TUTMa16	0.5	500	0	0	2.05 ± 0.24
TUTMa17	0.5	500	0	0	3.53 ± 0.18
TUTMa18	0.01	50	75	0	1.52 ± 0.29
TUTMa19	0.01	50	0	0	0
TUTMa20	3	50	0	0	0
TUTMa21	0.5	50	0	0	0
TUTMa22	1	0	0	0	0
TUTMa23	0.01	0	0	0	0
TUTMa24	0.5	500	0	0	0
TUTMa25	3	0	0	0	0
TUTMa26	0.01	0	0	0	0
TUTMa27	0.5	500	0	0	0.68 ± 0.02
TUTMa28	3	500	0	30	69.71 ± 2.74
TUTMa29	0.01	0	0	0	0.95 ± 0.18
TUTMa30	0.01	0	0	0	0
TUTMa31	3	0	0	50	35.98 ± 5.32
TUTMa32	0.01	0	0	0	0
TUTMa33	0.5	500	0	0	0
TUTMa34	0.01	0	0	0	0.40 ± 0.21
TUTMa35	0.01	500	0	0	0.30 ± 0.02
TUTMa36	7	500	150	0	0
TUTMa37	7	500	0	0	0
TUTMa38	0.01	0	0	0	25.26 ± 4.08
TUTMa39	0.01	500	0	0	0
TUTMa40	0.01	0	0	0	0.072 ± 0.02
TUTMa41	0.5	500	0	0	0
TUTMa42	0.5	500	0	0	0
TUTMa43	3	500	0	40	7.26 ± 0.08
TUTMa44	3	500	0	0	15.60 ± 0.05
TUTMa45	0.01	0	0	0	0
TUTMa46	0.01	0	0	0	0
TUTMa47	0.5	500	0	0	0
TUTMa48	0.01	0	0	0	2.40 ± 0.13
TUTMa49	0.01	0	0	0	0
TUTMa50	0.01	0	75	0	0
TUTMa51	0.01	0	0	0	0
TUTMa52	0.01	0	0	0	0.02 ± 0.05
TUTMa53	0.01	0	0	0	0.32 ± 0.21
TUTMa54	0.01	0	0	0	0
TUTMa55	0.01	0	150	0	1.80 ± 0.05
TUTMa56	0.01	0	150	0	1.10 ± 0.18
TUTMa57	0.01	0	150	0	1.65 ± 0.43
TUTMa58	0.01	0	0	0	1.31 ± 0.10
TUTMa59	0.01	0	0	0	0.05 ± 0.05
TUTMa60	0.01	0	0	0	1.50 ± 0.16
TUTMa61	0.01	0	0	0	0
TUTNa62	0.01	0	75	0	2.03 ± 0.21
TUTNa63	0.01	0	75	0	0
TUTNou64	0.01	0	75	0	0
TUTNou65	0.01	0	0	0	26.06 ± 0.21
TUTNou66	0.01	0	150	0	24.62 ± 0.21
TUTNou67	0.01	0	0	0	35.55 ± 0.62
TUTNou68	7	0	75	60	6.02 ± 0.16
TUTNou69	0.01	0	75	0	22.13 ± 0.02
TUTNou70	0.01	0	0	0	0
TUTNou71	7	0	25	100	4.05 ± 0.05
TUTNou72	0.01	0	0	0	0
TUTNou73	3	0	0	20	0
TUTNou74	0.01	0	150	0	0
TUTNou75	0.01	0	75	0	0
TUTNou76	0.01	0	25	0	0
TUTKa77	0.01	0	0	0	0
TUTKa78	0.01	0	0	0	0
TUTKa79	5	500	150	20	1.54 ± 0.05
TUTKa80	0.01	0	0	0	0
TUTKa81	2	100	75	0	2.87 ± 0.05
TUTKa82	0.01	0	0	0	0
TUTGh83	0.01	0	0	0	9.01 ± 0.13
TUTGh84	0.01	0	150	0	0
TUTGh85	0.01	0	0	0	0
TUTGh87	0.01	0	0	0	21.71 ± 0.13
TUTGh88	0.01	0	0	0	0
TUTGh89	0.01	0	0	0	0
TUTGh90	7	100	75	0	0

### Intrinsic antibiotic resistance

A number of isolates were tolerant to high concentrations of the test antibiotics (streptomycin and kanamycin), while others were susceptible. Twenty four (24) isolates tolerated up to 500 µg.ml^−1^ streptomycin, while three of the isolates grew well in 100 µg.ml^−1^ concentration of streptomycin and four in 50 µg.ml^−1^ streptomycin. Eight isolates tolerated 150 µg.ml^−1^ kanamycin, and included TUTMa36, TUTMa55, TUTMa56 and TUTMa57 from South Africa, TUTNou66, TUTNou74 and TUTKa79 from Mali, and isolate TUTGh84 from Ghana. Moreover, only 10 isolates grew in 75 µg.ml^−1^ kanamycin while two grew in 25 µg.ml^−1^ of the antibiotic (Table 2).

### Screening for phosphate-solubilizing bacteria (PSB)

Phosphate-solubilizing bacteria are characteristically identified by the formation of a clear halo around their colonies due to phosphate solubilization on double layered plates of B3 media (basal layer) and tri-calcium phosphate (top layer). Here, the results showed that seven out of the 89 rhizobial isolates were able to solubilize tricalcium phosphate. However, the seven phosphate solubilizing bacteria differed in their ability to solubilize phosphate, as there was a difference in the diameter of the halo-zones surrounding each colony (Table 2). The diameter of the clear zone ranged from 20 to 100 mm, with isolates TUTNou68 and TUTNou71 exhibiting greater P-solubilizing ability than the other isolates (Table 2). In contrast, isolates TUTNou73 and TUTNou79 recorded the least P solubilization (20 mm) (Table 2).

### Indole acetic acid production

The isolates showed marked differences in their ability to produce IAA in tryptophan supplemented YM broth media. Of the 89 isolates, 39% (35 isolates) produced a detectable amount of IAA, which ranged from 0.02 to 69.71 µg.ml^−1^, while the auxin levels produced by the remaining 54 isolates were below the detection limit. A large amount of IAA was produced by isolate TUTMa28, followed by isolates TUTMa31, TUTNou67, TUTNou65 and TUTNou66, while the lowest level was by isolate TUTMa52 (Table 2).

### ERIC-PCR amplification

**Figure 2:**
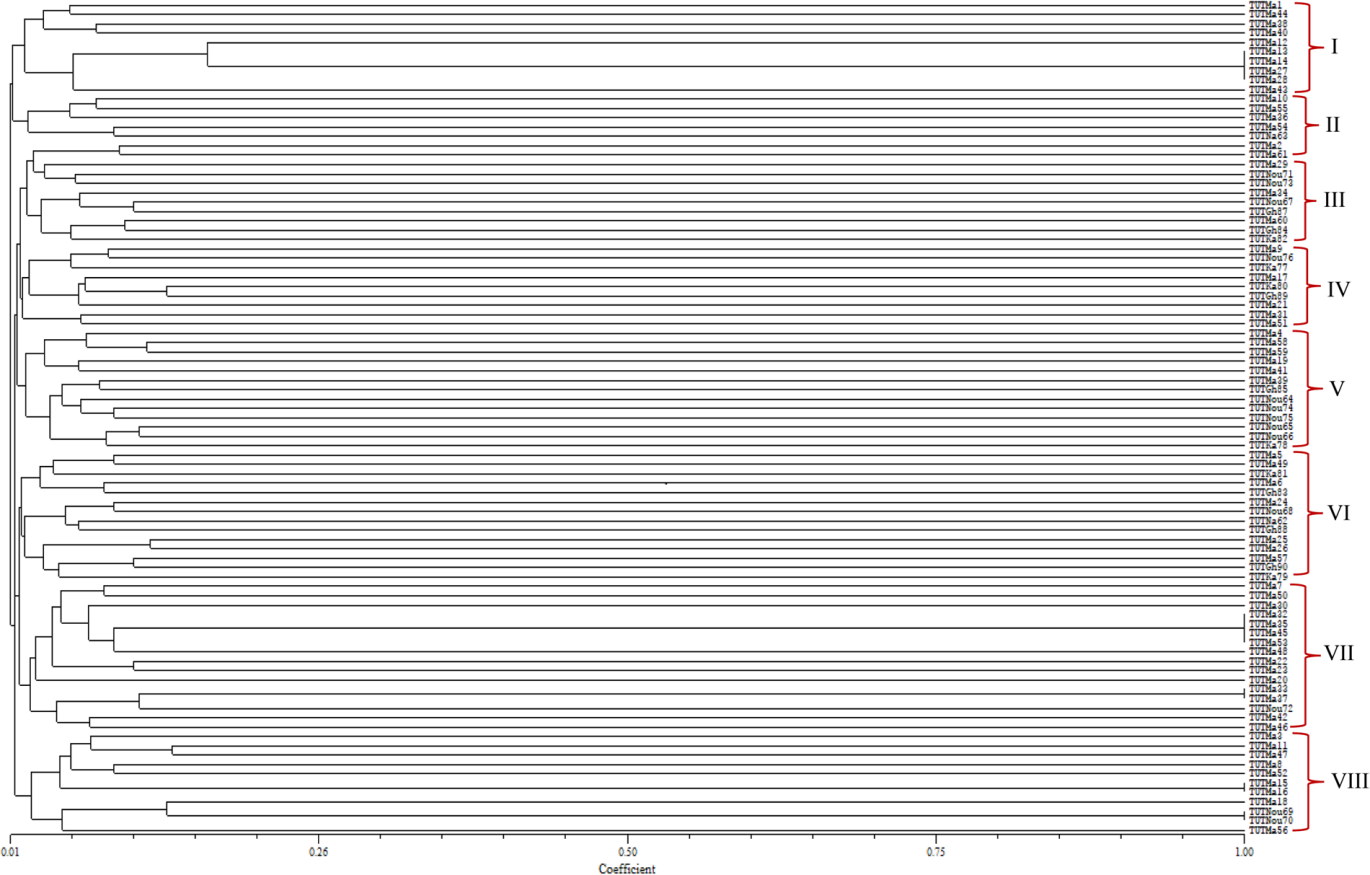
A dendrogram generated from ERIC-PCR banding patterns of 89 Bambara groundnut rhizobial isolates. The lengths of the separated DNA amplified fragments on the gel were calculated using Image Lab software (Bio Rad, USA). The fragment length was used to construct a dendrogram using Jaccard’s similarity coefficient with NTSYSpc 2.1 software.

PCR amplification of the ERIC region of the genomic DNA from each isolate yielded distinctive banding patterns. Isolates TUTMa17, TUTMa46 and TUTMa59 recorded the maximum number of bands (23 bands), while isolate TUTMa5 produced the minimum number of five bands. Most of the bands were clear, except for a few faint ones. The dendrogram generated from the banding patterns placed the isolates into eight major clusters (Fig. 2). The first cluster (Cluster I) contained 10 isolates from South Africa, with TUTMa13, TUTMa14, TUTMa27 and TUTMa28 showing a high Jaccard’s similarity coefficient of 1.00. Cluster II comprised five isolates, which comprised four from South Africa and one from Mali (TUTNa63). Clusters III, IV, V and VI contained isolates of mixed geographic origin and respectively comprised 11, 9, 13 and 14 isolates from all three studied countries (i.e. Ghana, Mali and South Africa). Sixteen isolates were grouped together in Cluster VII and included one isolate from Mali (TUTNou72) and 15 from South Africa. Cluster VIII contained 11 isolates which included two from Mali (TUTNou69 and TUTNou70) and the remainder from South Africa (Fig. 2). Overall, it was interesting to note that isolates which showed Jaccard’s similarity coefficient of 1.00 were all from the same location. Isolates from Ghana did not cluster together or with any other isolates at 1.00 Jaccard’s similarity coefficient (Fig. 2).

### Influence of soil properties on bradyrhizobial distribution

**Figure 3:**
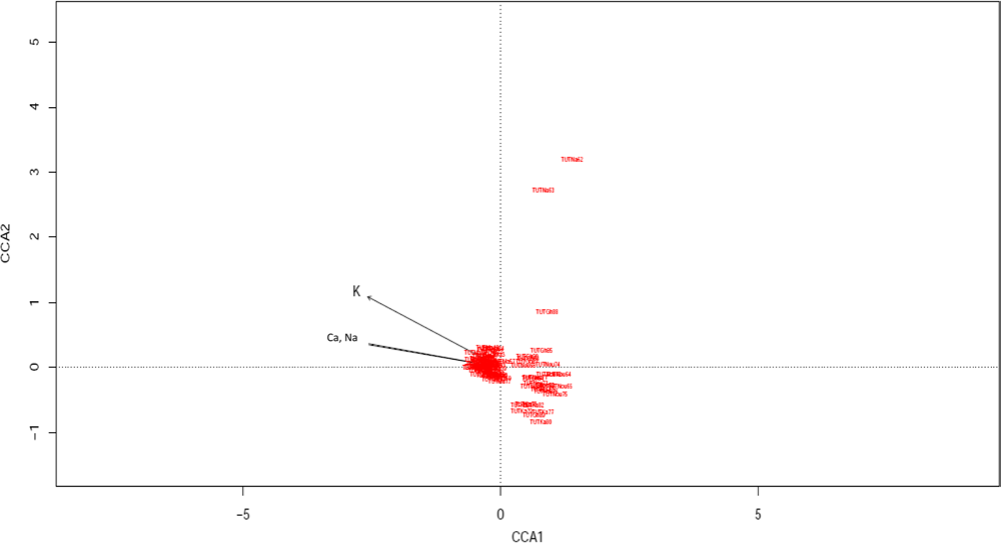
Canonical correspondence analysis (CCA) plot generated using the data from ERIC-PCR banding pattern and soil chemical properties to show the distribution of Bambara groundnut nodulating rhizobia from different locations in Mali, South Africa and Ghana.

In this study, the concentration of soil Ca, Na and K showed significant (p ≤ 0.01) effect on rhizobial distribution (Fig. 3). The soil pH, K and P were not included in the graph because of their insignificant effect on the distribution of bradyrhizobial populations in the soils studied. In the canonical correspondence analysis (CCA) ordination plot, the total mean square contingency coefficient (inertia) was 13.38 which consisted of 12.78 unconstrained (unexplainable) and 0.59 constrained (explainable) variables. The eigen analysis of constrained variables indicated that the proportion of species variance explained by the first axis (CCA1) was 42.4%, while the second axis (CCA2) explained 33.9% of the variance (Fig. 3). The ordination plot showed that the concentration of Ca and Na in soil had similar correlations with the first canonical axis (CCA1) just as soil K was correlated with the same axis (CCA1). All the test isolates from Marapyane (South Africa) were closely grouped at the centre of the CCA ordination plot, while the isolates obtained from the other locations in Ghana and Mali grouped separately from the centre of the ordination, indicating the effects of geographic location of the distribution patterns of the isolates (Fig. 3).

### Phylogenetic analysis of the 16S-rRNA gene

The PCR-amplified products of 1500 bp from the 16S-rRNA genomic region of the isolates selected from clusters of ERIC-PCR results were used for sequencing and phylogenetic analysis. The maximum likelihood phylogeny of the 16S-rRNA gene grouped the 25 selected isolates into four distinct clades within the genus *Bradyrhizobium* (Supplementary Fig. S1).

### Sequence and phylogenetic analyses of housekeeping genes (*atpD*, *glnII*, *gyrB* and *recA*)

For a robust phylogenetic analysis, four conserved housekeeping genes (*atpD*, *glnII*, *gyrB* and *recA*) were selected and studied. The PCR amplification of the housekeeping genes *atpD*, *glnII*, *gyrB*, and *recA* yielded amplified products comprising single bands of approximately 600 bp, 650 bp, 700 bp, and 600 bp, respectively. The sequences used in the phylogenetic analysis of each gene are described in Supplementary Table S1. The maximum likelihood phylogenetic trees placed the isolates in five distinct clades within the genus *Bradyrhizobium* (Supplementary Figs S2–S5). Except the position of isolate TUTNou66 in *glnII* phylogeny, the topography of the individual (*atpD*, *glnII*, *gyrB* and *recA*) housekeeping gene phylogenies were very similar and consistent with the 16S rRNA phylogeny (Supplementary Figs S1–S5). Isolate TUTNou66 obtained from Mali clustered with other isolates (TUTNou64, TUTNou69, TUTNou70, TUTNou74, TUTNou76) from Mali in Clade III in the *atpD* phylogeny and grouped with the South African isolate TUTMa54 in Clade II in the *gyrB* and *recA* phylogenies, but stood separately in the *glnII* phylogeny.

### Phylogenetic position of the isolates based on concatenated *atpD*, *glnII*, *gyrB* and *recA* genes sequence analysis

**Figure 4:**
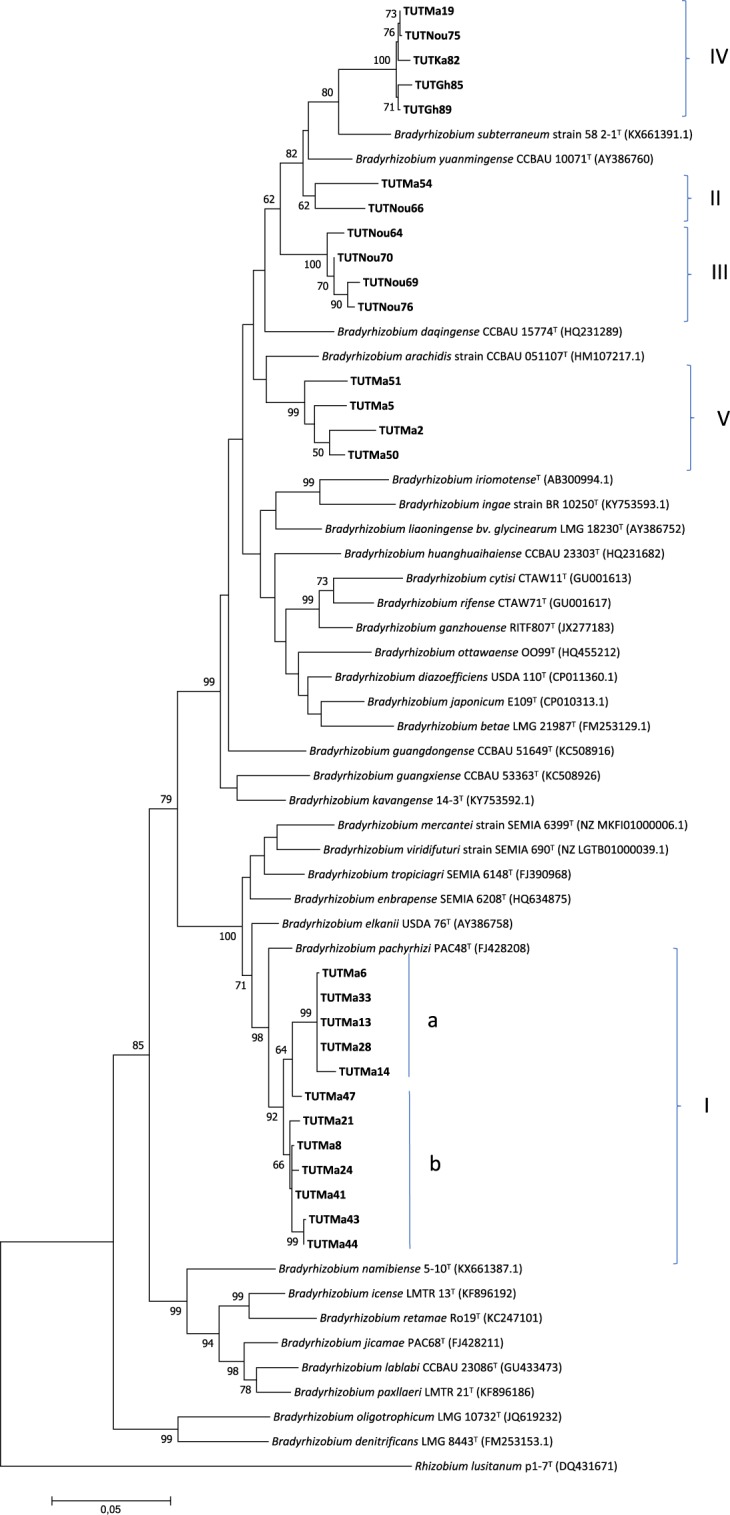
Phylogenetic tree based on *atp*D-*gln*II*-gyr*B*-rec*A genes sequences generated by Maximum likelihood method. Bootstrap values (1000 replicates) are indicated above the branches which shows the percentage of trees in which the associated taxa clustered together. The number of nucleotide substitution is indicated by scale bar. All missing and gap nucleotide sequences were eliminated during phylogenetic tree construction. Evolutionary analyses were conducted in MEGA7.

To generate a more robust phylogenetic tree of the tested isolates within the genus *Bradyrhizobium*, a concatenated *atpD* + *glnII* + *gyrB* + *recA* gene phylogenetic analysis was performed. Due to the absence of some isolates in the single housekeeping gene phylogenies, only 27 isolates were used in the concatenated sequence analysis. The consensus sequence length was 1428 bp and comprised 827 conserved, 601 variables, 397 parsimony informative and 204 singleton sites (Supplementary Table S1). The maximum likelihood tree of the concatenated sequences was congruent with the single gene phylogenetic trees and grouped the isolates into five major clades (Clade I-V) (Fig. 4). The isolates from South Africa in Clade I formed two subgroups (Ia and Ib) and were proximally related to the reference type strain *Bradyrhizobium pachyrhizi* PAC48^T^ with 96.8 to 98.1% sequence identity and 98% bootstrap support (Fig. 4).

In Clade II, isolates TUTMa54 and TUTNou66 grouped together and stood separately from any reference type strains, though close to the reference type strain of *Bradyrhizobium yuanmingense*. The isolates in Clade III (TUTNou64, TUTNou69, TUTNou70 and TUTNou76) were all from Nougani in Mali and did not group with any reference type strain, but *Bradyrhizobium arachidis*, showed up as the closest related species to them with low sequence identity of 94.8 to 95.4%. Isolates TUTMa19, TUTNou75, TUTKa82, TUTGh85 and TUTGh89 in Clade IV grouped together and were proximally related to *Bradyrhizobium subterraneum* with 80% bootstrap support and 95.1 to 95.5% sequence similarity. Four isolates from South Africa, (TUTMa51, TUTMa5, TUTMa2 and TUTMa50) grouped together and stood separately in Clade V without any close reference type strains (Fig. 4).

### Isolates’ phylogenetic position based on *nifH* and *nodC* genes

**Figure 5:**
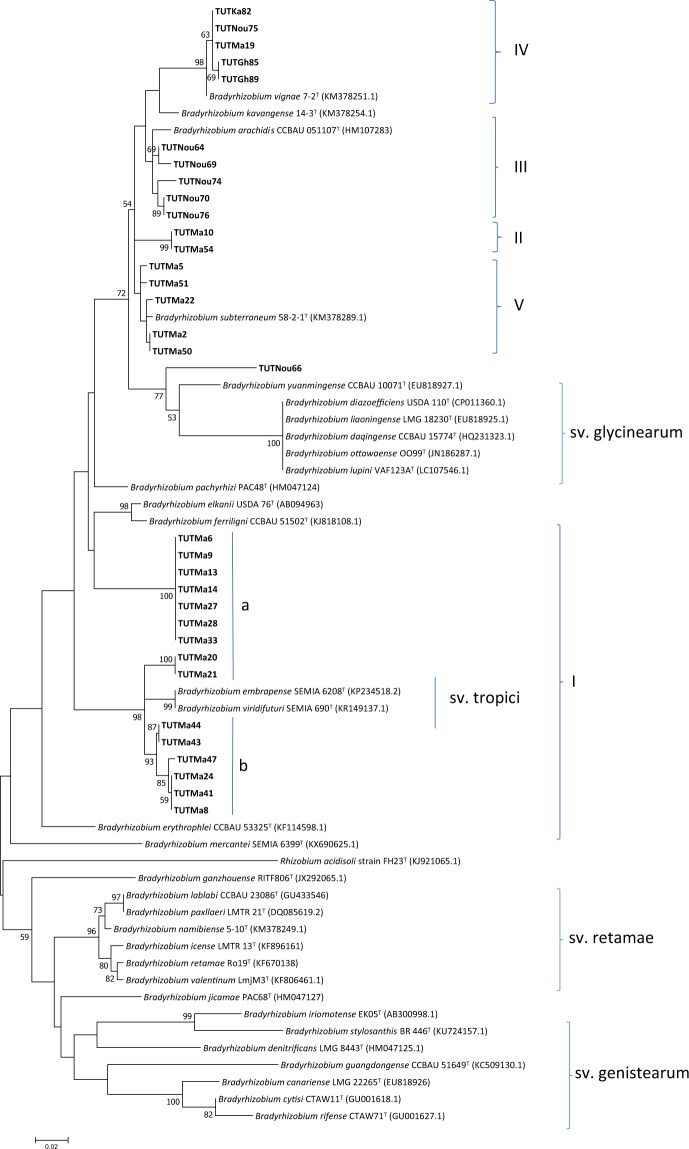
Phylogenetic tree based on *nifH* gene sequences generated by Maximum likelihood method. Bootstrap values (1000 replicates) are indicated above the branches which shows the percentage of trees in which the associated taxa clustered together. The number of nucleotide substitution is indicated by scale bar. All missing and gap nucleotide sequences were eliminated during phylogenetic tree construction. Evolutionary analyses were conducted in MEGA7.

**Figure 6:**
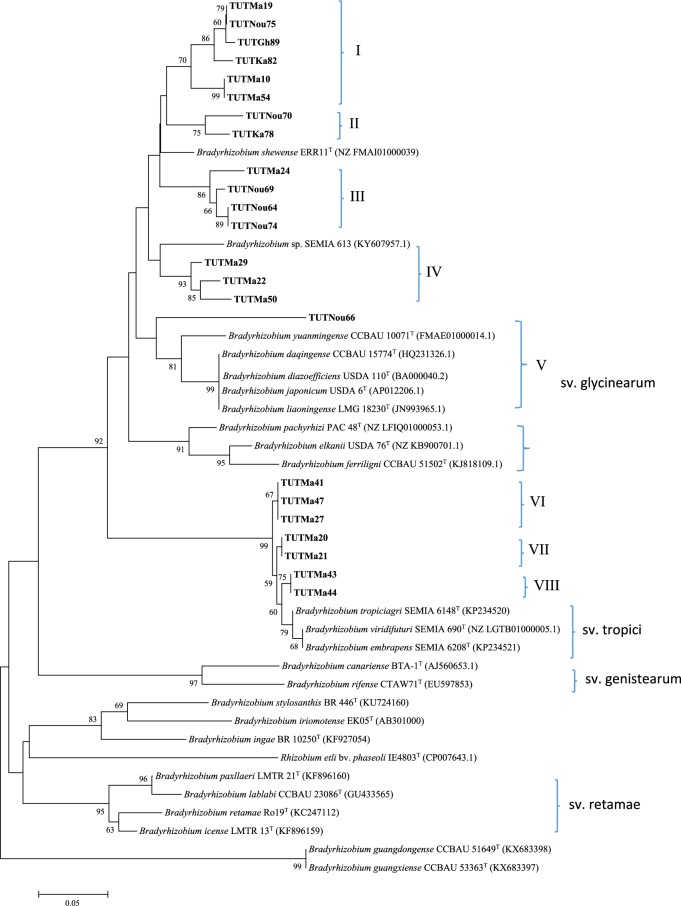
Phylogenetic tree based on *nodC* gene sequences generated by Maximum likelihood method. Bootstrap values (1000 replicates) are indicated above the branches which shows the percentage of trees in which the associated taxa clustered together. The number of nucleotide substitution is indicated by scale bar. All missing and gap nucleotide sequences were eliminated during phylogenetic tree construction. Evolutionary analyses were conducted in MEGA7.

The PCR-amplified products of *nifH* and *nodC* genes were 800 bp and 300 bp band sizes. Phylogenetic analyses of the *nifH* and *nodC* genes placed the tested isolates in various clades similar to the housekeeping gene phylograms, though with some inconsistencies between the phylogenies. Isolate TUTMa22 clustered with isolates TUTMa5, TUTMa51, TUTMa2 and TUTMa50 in Clade V of the *nifH* phylogeny, and with TUTMa50 in Clade IV of the *nodC* phylogeny but stood separately in the housekeeping gene phylogenies (Figs 5 and 6). Not all the test isolates showed close relationship with known reference strains. For example, the isolates in Clade I were distantly related to the reference type strains, but shared 92.7% sequence identity with *Bradyrhizobium elkanii* USDA 76 ^T^, the closest reference type strain, which was congruent with the single gene phylogenetic trees. Only isolate TUTNou66 showed a clear resolution with symbiovar glycinearum (Figs 5 and 6).

### Nodulation, symbiotic effectiveness and plant growth

**Figure 7:**
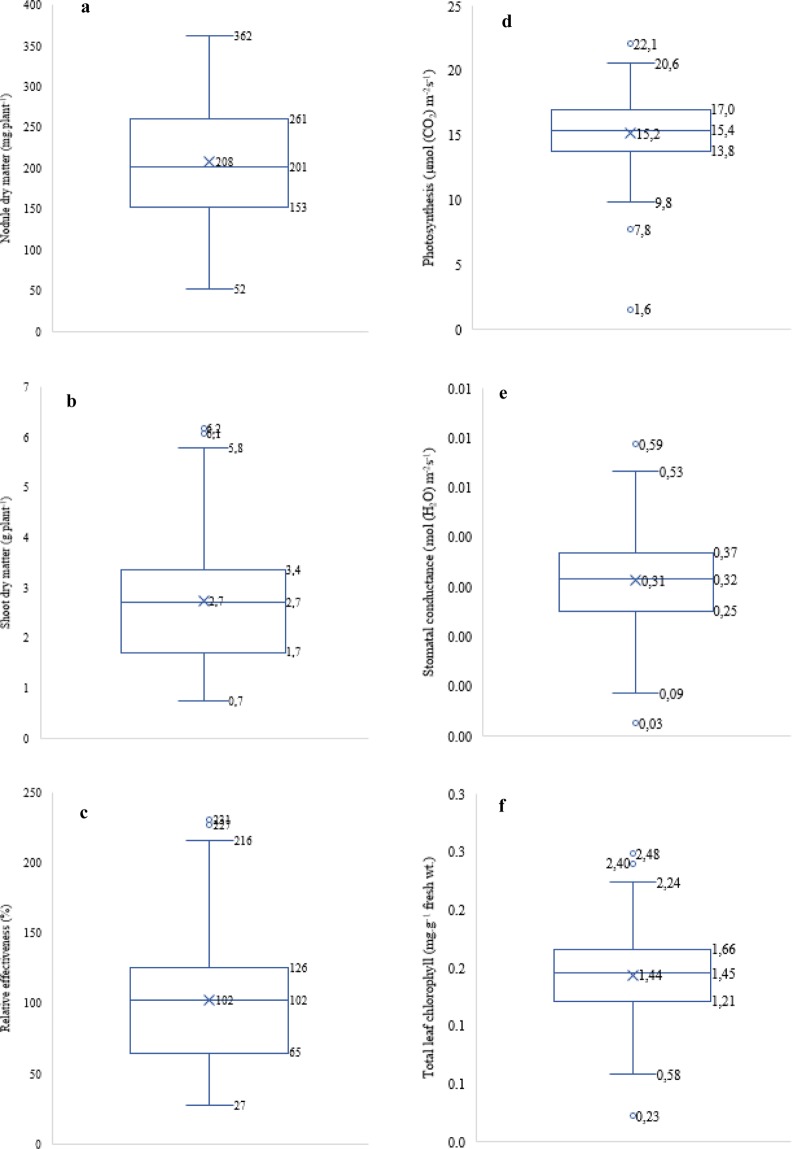
Box plots showing the distribution of (**a**) nodule dry matter, (**b**) shoot dry matter, (**c**) relative effectiveness, (**d**) photosynthetic rates, (**e**) stomatal conductance and (**f**) leaf transpiration rates induced by indigenous Bambara groundnut isolates from Ghana, South Africa and Mali. Centre lines show the medians; X indicates the mean; box limits indicate the lower (Q1) and upper (Q3) quartiles; whiskers extend 1.5 times the interquartile range from the lower and upper quartiles, outliers are represented by dots.

**Figure 8:**
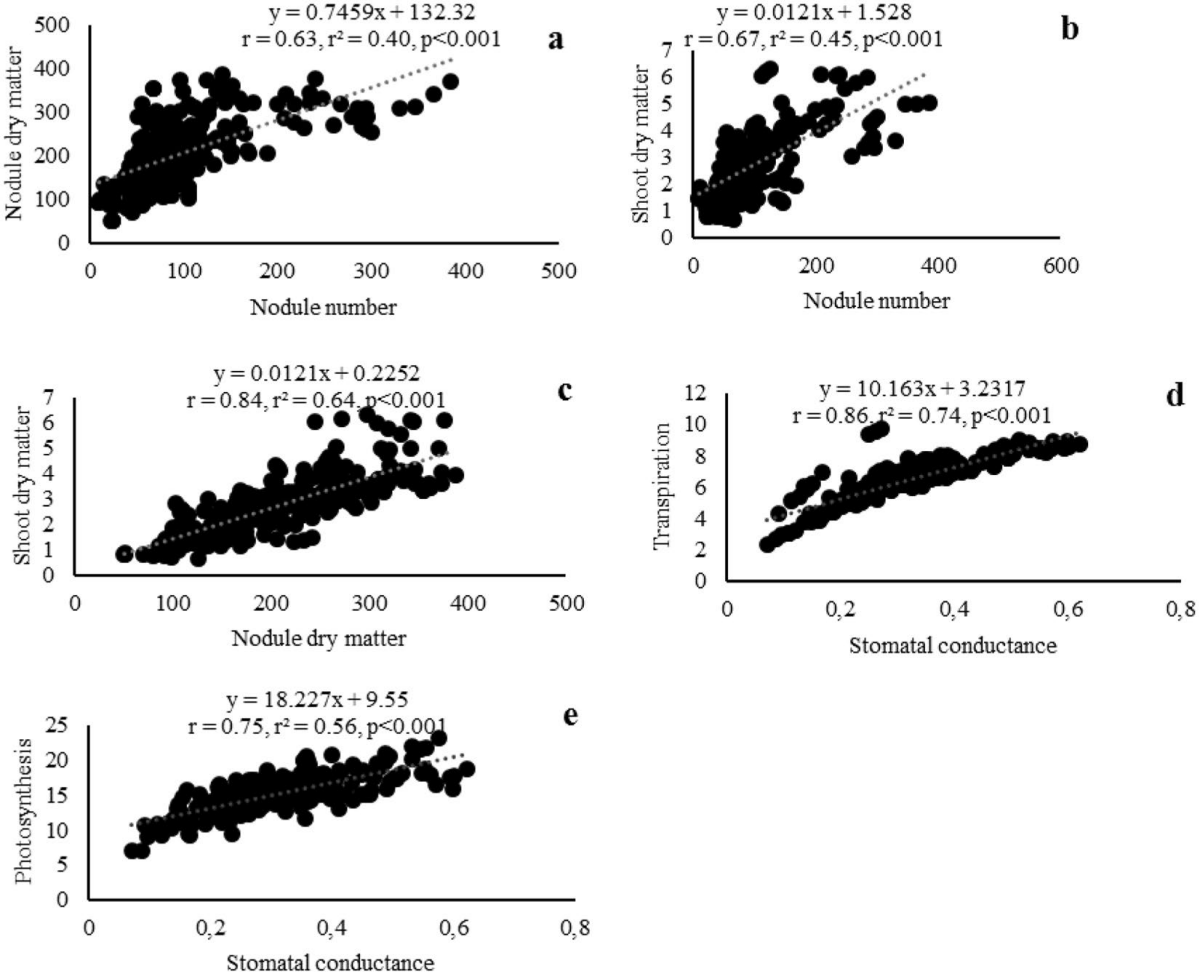
Correlation and regression analysis between (**a**) nodule number and nodule DM, (**b**) nodule number and shoot DM, (**c**) nodule DM and shoot DM, (**d**) stomatal conductance and transpiration and (**e**) stomatal conductance and photosynthesis.

The indigenous Bambara groundnut rhizobial isolates from the three African countries studied induced variable (p < 0.001) nodulation (nodule number and nodule dry matter) on their homologous host (Fig. 7; Supplementary Table S2). Isolate TUTKa80 produced the highest number of nodules, followed by isolates TUTNou67, TUTNa62 and TUTNou75, which all belonged to different ERIC-PCR clusters (Supplementary Table S2; Fig. 2). Of the 89 isolates tested, 45% induced more nodules (90–366 nodules per plant) on Bambara groundnut than the commercial *Bradyrhizobium* strain CB756 (70 nodules per plant) (Supplementary Table S2). Generally, the rhizobia that induced higher nodule numbers, also elicited greater nodule dry matter in the Bambara groundnut host plant, albeit a few exceptions (Fig. 8a). The nodule mass produced by the test isolates ranged from 52 mg.plant^−1^ for isolate TUTGh83 to 362 mg.plant^−1^ by isolate TUTMa11 (Fig. 7; Supplementary Table S2). About 74% of the isolates induced greater nodule DM (160–362 mg.plant^−1^) than the commercial strain CB756 which produced 122 mg.plant^−1^, a value close to the lower quartile (Fig. 7; Supplementary Table S2).

As found with nodulation, shoot DM yield of host plant differed significantly among test isolates, with values ranging from 0.73 g.plant^−1^ for isolate TUTMa36 to 6.2 g.plant^−1^ for TUTMa21 (Fig. 7; Supplementary Table S2). In general, an increase in nodule number and/or nodule dry matter were accompanied by higher shoot biomass (Fig. 8b,c). Furthermore, 66% of the isolates tested induced greater shoot DM (2.2 to 6.2 g.plant^−1^) than the commercial *Bradyrhizobium* strain CB756 (1.9 g.plant^−1^). Also, 37% of the isolates produced greater shoot DM when compared to the 5 mM KNO_3_-fed plants, which recorded shoot biomass (2.7 g.plant^−1^) similar to the mean and median values of the dataset (Fig. 7; Supplementary Table S2). Together with the uninoculated control, the plants inoculated with isolates TUTMa33, TUTMa36 and TUTGh83 produced the lowest shoot DM with a range of 0.7 to 1.0 g.plant^−1^ (Supplementary Table S2).

The observed differences in nodulation and plant dry weight yield were accompanied by marked variations in the relative symbiotic effectiveness of the test isolates, which ranged from 27% for isolate TUTMa36 to 231% for TUTMa21 (Fig. 7; Supplementary Table S2). Of the 89 isolates tested, 66% were significantly more effective (84–231% REi) than the commercial *Bradyrhizobium* strain CB756, which had a REi of 70% (Supplementary Table S2). Based on shoot DM, 11% of the isolates were classified as low N_2_-fixers (i.e. <50% RE), while 20% of the isolates, including the commercial strain CB756, were moderately effective (50–80% REi) (Supplementary Table S2). The remaining isolates were scored as highly effective (>80% REi).

### Photosynthesis rates as a measure of isolate relative effectiveness

Inoculating Bambara groundnut seedlings with the test isolates induced varying leaf photosynthetic rates, stomatal conductance and chlorophyll concentrations, which contributed to the differences in symbiotic effectiveness (Fig. 7; Supplementary Table S2). There were marked variations in the photosynthetic rates induced by the test isolates, with values ranging from 9.9 µmol (CO_2_) m^−2^s^−1^ for isolate TUTMa53 to 22.1 µmol (CO_2_) m^−2^s^−1^ for isolate TUTNou60 (Fig. 7; Supplementary Table S2). As to be expected, increased photosynthetic rates were generally accompanied by higher stomatal conductance and leaf transpiration rates (Figs 8d,e). The lowest chlorophyll concentrations were found in the uninoculated control and the 5 mM KNO_3_-fed plants, which expectedly recorded the least stomatal conductance and smaller photosynthetic rates of 1.6 and 7.8 µmol (CO_2_) m^−2^s^−1^, respectively (Fig. 7; Supplementary Table S2).

## Discussion

### Morpho-genetic diversity of native Bambara groundnut-nodulating symbionts and the effect of soil factors on their distribution

This study described the biodiversity, phylogenetic relationships and the symbiotic effectiveness of microsymbionts nodulating Bambara groundnut, a nutritionally important but underutilized African legume. Of a total of 201 bacterial isolates tested from Ghana, Mali and South Africa, 44% (89 isolates) were able to induce effective nodulation on their homologous host, while six others formed ineffective nodules. The presence of non-nodulating endophytes in root nodules of Bambara groundnut as observed in this study was earlier reported for soybean^23^ and *Vigna* species^22,24^. The few non-functional nodules observed here could be pseudonodules formed due to factors that may include the perception of nod factors released by non-invasive rhizobia^25,26^. The 89 rhizobial isolates were found to exhibit high morpho-physiological diversity, which ranged from differences in colony size, shape and texture to growth rates when cultured on YMA plates. Earlier reports by Grönemeyer *et al*.^8^, Ngo Nkot *et al*.^27^ and Benson *et al*.^12^ also found that Bambara groundnut was nodulated by both slow- and fast-growing *Bradyrhizobium* types. Analysis of the ERIC-PCR profiles further revealed high genomic diversity among rhizobia nodulating Bambara groundnut in the soils studied, thus agreeing with previous reports which found high genetic diversity among microsymbionts of Bambara groundnut in other African soils^22^.

In present study, soil chemical properties influenced the distribution of the rhizobial isolates obtained from South African, Ghanaian and Malian soils. The South African isolates which showed close relatedness to *B*. *elkanii* and *B*. *yuanmingense* in the phylogenetic analysis were positioned at the centre of the ordination plot and were greatly influenced by the soil Ca and Na levels, in contrast to the mild effect of K on the distribution of the same isolates. The effect of soil Na concentration on the distribution of *B*. *elkanii* was recently reported by Mohammed *et al*.^13^. Similarly, the soil concentration of K has been reported to alter the distribution of rhizobia in African and Chinese soils^3,28^. Puozaa *et al*.^3,19^ and Ndungu *et al*.^29^ also recently reported a significant influence of soil mineral nutrients on the bradyrhizobial distribution in some African soils, which all indicate the roles of these soil factors on the occurrence and persistence of rhizobial types in different geographic areas.

### Phylogenetic relationships of Bambara groundnut-nodulating symbionts in African soils

Various studies on the microsymbionts nodulating Bambara groundnut have shown that this native African legume is nodulated by species belonging to the genus *Bradyrhizobium* in Ghana, South Africa, Angola and Namibia^3,8^. In this study, the concatenated *atpD* + *glnII* + *gyrB* + *recA* phylogeny placed the isolates in five distinct groups which were highly divergent from the reference type strains, suggesting that these rhizobia probably represent novel species within the *Bradyrhizobium* genus. For example, isolates in Clades III, IV and V showed very low (94–95%) sequence identity with the reference *Bradyrhizobium* type strains. The single gene phylogenies of the test isolates were however highly congruent with each other and with the concatenated gene phylogeny. For example, this congruence was observed in the isolates that clustered with the *B*. *elkanii*, *B*. *pachyrhizi* and *B*. *vignae* reference type strains in the 16S rRNA and concatenated *atpD* + *glnII* + *gyrB* + *recA* gene phylogenies. Aside the observed consistency in clustering between housekeeping gene phylogenies, there was also congruency in clustering between the core and symbiotic gene phylogenies of some isolates, a finding that could suggest that their nodulation and symbiotic genes were maintained through vertical gene transfer. However, there were some isolates that showed incongruences in the housekeeping gene phylogenies, which could be attributed to possible intra-genomic rearrangements, horizontal gene transfer, and/or subsequent recombination events^3,22,30–33^. As found with the native symbionts of Bambara groundnut in this study, earlier studies have also reported the presence of potentially novel bradyrhizobia responsible for Bambara groundnut nodulation in different African soils^3,8^. However, the fact that this study provides a detailed report of Bambara groundnut nodulating symbionts from Mali suggests that future studies in that country could unravel novel species associated with the nodulation of this grain legume.

### Symbiotic effectiveness of Bambara groundnut-nodulating microsymbionts

Besides their reported high morpho-genetic diversity and distribution, the Bambara groundnut rhizobial isolates also varied in their N_2_-fixing effectiveness, a trait that is vital for their use as inoculants^13^. Screening rhizobial isolates for high symbiotic efficacy is a first step to determine their use as potential inoculant strains. In this study, the phylogenetically distinct isolates were found to exhibit marked variation in N_2_-fixing effectiveness and actual N_2_ fixation. This was illustrated by the significant differences in nodulation, shoot dry matter, leaf chlorophyll concentration and photosynthetic activities induced on Bambara groundnut by the rhizobial isolates tested. The increased and effective nodulation caused by the test isolates resulted in high leaf chlorophyll content, photosynthetic rates and shoot biomass when compared to the NO_3_^−^ fed plants. Probably because of nitrate leaching, even the relatively low biomass-inducing isolates elicited greater leaf chlorophyll concentrations and photosynthetic rates in the Bambara groundnut host than nitrate feeding (Supplementary Table S2). These findings led to significant correlations when nodule number and nodule dry matter were each plotted against plant growth (Fig. 8b,c). The increased photosynthetic functioning in most inoculated plants were also evidenced by a significant correlation obtained when leaf stomatal conductance and transpiration were each plotted against photosynthetic rates induced in the Bambara groundnut host (Fig. 8d,e). Other studies by Benson *et al*.^12^ Gyogluu *et al*.^34,35^ and Mohammed *et al*.^36^ also found that inoculating different legumes with N_2_-fixing native rhizobia from various African countries led to greater photosynthetic functioning and increased accumulation of biomass in glasshouse experiments. The fact that most isolates in this study (about 66%) exhibited higher N_2_-fixing efficiency than the commercial *Bradyrhizobium* strain CB756 suggests that these native rhizobial isolates of this legume are potential candidates for use as inoculants.

### Adaptive and plant-growth promoting traits of native rhizobial symbionts of Bambara groundnut

The N_2_-fixing effectiveness of rhizobia is a first step to their N contribution in cropping systems. But their adaptation to various stress factors such as soil antibiotics and salinity is important for their survival in the rhizosphere^11^. In this study, the highly effective native rhizobial isolates nodulating Bambara groundnut showed variations in their tolerance to different levels of salinity and antibiotics often produced by antagonistic soil microbes. The test isolates (TUTMa6, TUTMa9, TUTMa13, TUTMa14, TUTMa27, TUTMa28, TUTMa33, TUTMa41, TUTMa43 and TUTMa44) from South Africa that elicited tolerance to high concentrations of streptomycin (500 µg.ml^−1^) shared close relationship with the *Bradyrhizobium elkanii* group in the 16S- rRNA, *glnII*, *gyrB*, *atpD*, *recA* and *nifH* phylogenies. Yue Li *et al*.^37^ also reported streptomycin tolerance in strains of *B*. *elkanii* and *B*. *pachyrhizi*. Similarly, Ramırez-Bahena *et al*.^38^ found that *B*. *pachyrhizi* showed tolerance to several antibiotics, except for kanamycin to which it was susceptible. In this study, the test isolates which showed tolerance to kanamycin were highly divergent from the *B*. *elkanii* and *B*. *pachyrhizi* type strains. However, the isolates that from Nougani in Mali that were tolerant to kanamycin at concentrations of 75 and 150 µg.ml^−1^ clustered together in Clade III of the phylogenetic trees. These isolates included TUTNou64 TUTNou66, TUTNou69, TUTNou74 and TUTNou75, which were closely related to *Bradyrhizobium arachidis* and *Bradyrhizobium yuanmingense* in the housekeeping and *nifH* gene phylogenies. These findings are consistent with a report by Grönemeyer *et al*.^39^ which found that *B*. *arachidis* and *B*. *yuanmingense* strains showed resistance to 20 µg.ml^−1^ of kanamycin. According to Bedi and Naglot^40^, intrinsic antibiotic resistance in rhizobia is linked to the presence of these antibiotics in the soils of bacterial origin. The resistance of rhizobia to antibiotics as observed for some isolates in this study is a trait that might contribute to their competitive and survival advantage in soils^9^. The isolates from Ghana were susceptible to the concentrations of antibiotics used in this study, suggesting that they were probably not previously exposed to these antibiotics in their natural environments. The observed variations in rhizobial tolerance to salinity and antibiotics in this study could influence their persistence and performance in different environments that may be characterised by those stress factors.

In addition to their resistance to antibiotics, some isolates from Mali and South Africa, (e.g. TUTMa28, TUTMa43, TUTMa44, TUTNou66 and TUTNou69) also produced substantial levels of indole acetic acid, a plant-growth promoting auxin. Of the isolates tested, TUTMa28 produced the highest concentration of IAA (69.71 µg.ml^−1^) and was closely related to the *B*. *elkanii* and *B*. *pachyrhizi* reference strains in the phylogenies of 16S-rRNA, *atpD*, *glnII*, *gyrB*, *recA* and *nifH* genes. Other studies have similarly found high IAA-producing isolates that grouped with *B*. *elkanii*^41,42^. The site of origin of the rhizobial isolates seem to have influenced their ability to solubilize P (tricalcium phosphate) or tolerate sodium chloride (NaCl) in the culture media. For example, although salt tolerant isolates could be found in most of the locations tested, majority of those isolates originated from the saline soils of Marapyane in South Africa, just as isolates TUTNou68 and TUTNou71 from the low P soils of Nougani were able to solubilize more tricalcium phosphate than their counterparts from the other locations with relatively higher levels of soil P. However, isolates TUTMa28, TUTMa31, TUTMa43, TUTNou68, TUTNou71, TUTNou73 and TUTKa79 which showed tolerance to 3–7% NaCl were also able to solubilize tricalcium phosphate. These results are consistent with those of Ngo Nkot *et al*.^27^ who found that rhizobial symbionts of Bambara groundnut were capable of growing in culture media supplemented with up to 4% NaCl. Whereas the observed ability of Bambara groundnut nodulating isolates to grow at high concentrations of NaCl (up to 7%) may improve their persistence in saline soils, their P solubilizing ability may contribute to plant-growth promotion in soils with low P availability. The size of the halo-zone produced by the PSB in this study ranged from 20 mm to 100 mm, and were within the range of values reported by Traoré *et al*.^43^ but higher than those reported by Yulianti and Rakhmawati^44^.

## Conclusion

The morpho-genetically diverse rhizobia isolated from Bambara groundnut grown in different geographic regions of Africa were also found to tolerate various levels of salinity and antibiotics. These isolates also differed in their ability to solubilize P and/or produce IAA, thus conferring varying ability to promote plant growth. The isolates from South Africa, Ghana and Mali were closely related to *B*. *vignae*, *B*. *subterraneum*, *B*. *kavangense*, *B*. *arachidis*, *B*. *yuanmingense*, *B*. *embrapense*, *B*. *pachyrhizi*, *B*. *elkanii* and *B*. *ferriligni*. However, further studies are required to determine the phylogenetic position of the isolates whose relationships were unclear and failed to group with any reference type strains. As the study of isolate effectiveness was conducted under glasshouse conditions, it is recommended that these strains be tested under field experiments to confirm rhizobial candidates for inoculant production.

## Materials and Methods

### Root nodule collection and soil sampling

**Figure 9:**
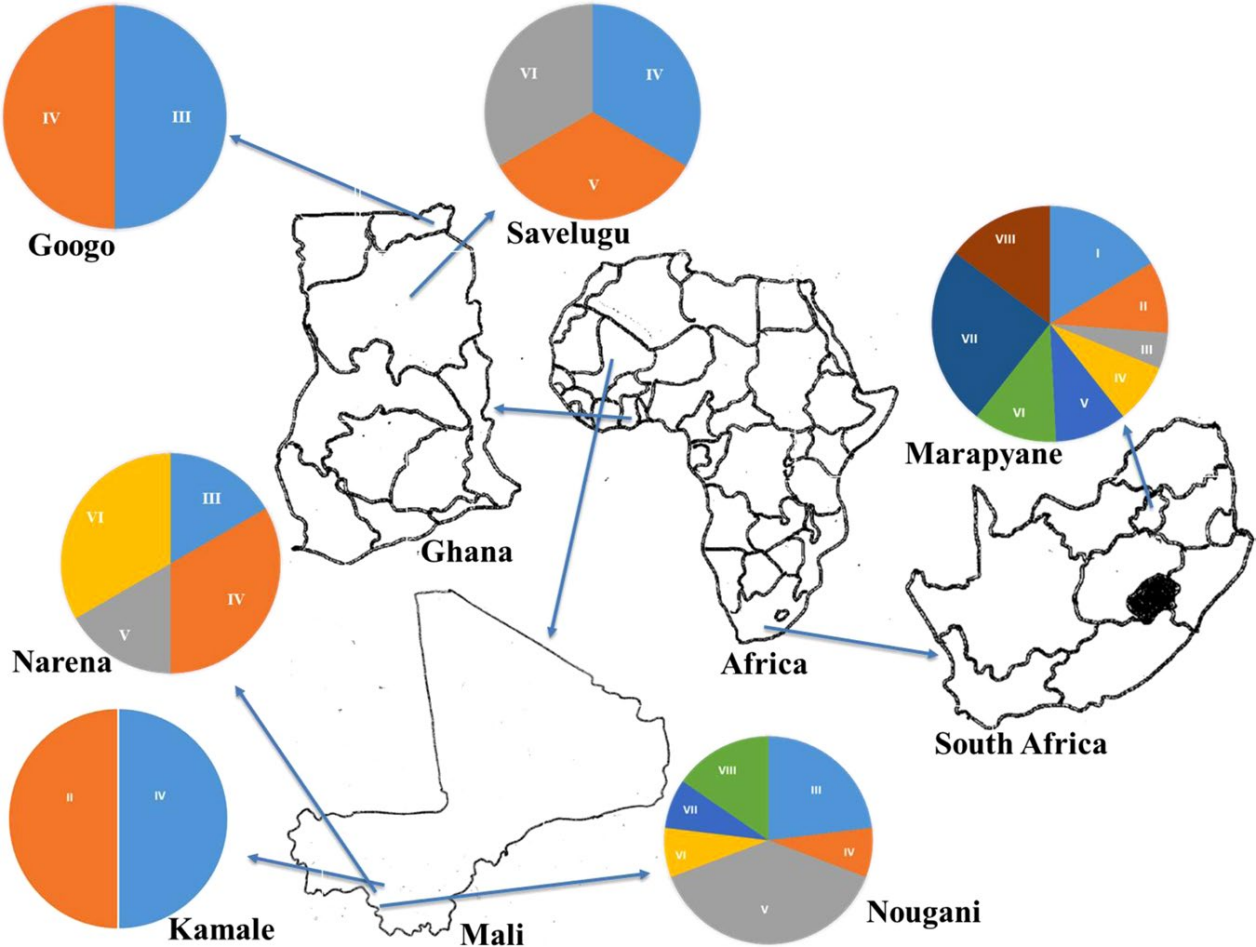
Geographical locations of each of the isolates. Pie graph of each location shows the number of isolates that belong to a particular ERIC-PCR cluster. Uppercase letters in pie graph represent the labels of ERIC-PCR clusters (see Fig. 3). The area of each segment is proportional to the number of isolates from a given location occupying that cluster.

The root nodules of Bambara groundnut used in this study were collected from farmers’ fields in Mali [Kamale (12°17′39″N, 8° 53′26″W), Narena (12°13′49″N, 8°37′59”W) and Nougani (12°1′0″N, 8°43′30″W)] in Mali, and from experimental plots in Ghana [Savelugu (9° 34′8.4″N, 0°49′47.99″W) and Googo (10°40′20.78″N, 0° 29′16.77″W)], as well as from South Africa [Marapyane (24°51′39.492″S, 28°50′20.544″E)] (Fig. 9). At each location, soil samples were collected at a depth of 0 to 20 cm and analysed for pH (H_2_0), Ca, K, P and Na using the Bray-2 method^45^.

### Isolation and authentication of root-nodule bacterial isolates

The collected root nodules were washed thoroughly under running tap to remove any debris. Each nodule was cut to retain a piece of root 0.5 cm long, and stored in silica gel for later use in bacterial isolation^46^. The nodule bacteria were isolated on yeast mannitol agar (YMA) plates, as described by Naamala *et al*.^32^. The plates were observed daily to record the number of days taken for colonies to appear.

The nodulation ability of the isolates was tested by using them to inoculate seedlings of Bambara groundnut as the homologous host. Bambara groundnut seeds were surface-sterilized with 70% ethyl alcohol for 2 to 3 minutes, washed with 3.5% NaOCl (bleach) for 2 to 3 minutes and then thoroughly rinsed (6 times) with sterile distilled water^46^. Pre-germinated Bambara seeds were transplanted in sterilized plastic pots (1.2 dm^3^) containing autoclaved sand. The Bambara groundnut seedlings were inoculated with 1 mL of yeast mannitol broth culture (10^7^ to 10^8^ rhizobial cells.ml^−1^) under axenic conditions. The pots with seedlings were then transported to the glasshouse and left to grow under natural conditions. Three replicate pots were used for each isolate and the plants were watered twice a week with N-free Dilworth nutrient solution^47^. Three pots with N-free uninoculated plants served as negative control, while 5 mM nitrate-fed plants and plants inoculated with the commercial inoculant (*Bradyrhizobium* sp. CB756) served as positive controls. Plants were uprooted from pot at 50 days after planting and assessed for nodulation.

### Symbiotic effectiveness of isolates and gas-exchange measurements of their nodulated plants

The rhizobial isolates were assessed for their symbiotic effectiveness in the glasshouse by measuring shoot dry matter (DM) and photosynthetic rates of seedlings. Leaf photosynthetic rates (A) and stomatal conductance (gs) of Bambara groundnut seedlings at 50 days after planting (DAP) were recorded on young and fully expanded green leaves using a portable infrared red gas analyzer, version 6.2 (LI 6400XT, Lincoln, Nebraska, USA). These gas-exchange measurements were done using the following chamber conditions: photosynthetic flux density of 1000 μmolm^−2^ s^−1^, reference CO_2_ concentration of 400 μmolmol^−1^ and flow rate of 500 μmols^−1^ ^13^. The leaves used for gas-exchange measurements were subsequently removed from the plants to determine chlorophyll formation and accumulation induced by the isolates. Three (3) green leaf discs (each with an area of 0.79 cm^2^ and weighing ≈ 17.8 mg) were taken to extract chlorophyll using preheated (65 °C) dimethyl sulfoxide (DMSO) for 30 mins. Before measuring absorbance, the spectrophotometer was calibrated to zero absorbance using pure DMSO and the absorbance of samples measured at 645 and 663 nm on a Jenway 7300 spectrophotometer. The amount of the total chlorophyll concentration was calculated by the equations described by Richardson *et al*.^48^. After photosynthesis measurement, plants were uprooted and data recorded for nodule number, nodule dry matter (nodule DM) and shoot dry matter (shoot DM) after oven-drying at 65 °C for 72 h. Shoot dry matter yield was used to calculate the relative symbiotic effectiveness (SEi) by comparing the biomass of each inoculated plant with the biomass of N-fed control plants. Relative symbiotic efficiency was estimated as Chibeba *et al*.^49^.

$$SEi=(\frac{{\rm{x}}}{{\rm{y}}})\ast 100$$

where x = dry matter of the inoculated plants and y = dry matter of plants supplied with 5 mM KNO_3_.

### Morpho-physiological characterization of rhizobial isolates

For phenotypic characterization, single colony cultures were re-streaked on YMA plates, incubated at 28 ± 2 °C and inspected daily for colony growth (number of days to colony appearance). The isolates were scored as slow-growers (>5 days), intermediate growers (4 to 5 days) or fast-growers (<4 days). Furthermore, the colony shape, colour, texture and size/diameter were also scored for each rhizobial isolate.

### NaCl tolerance of isolates

The ability of isolates to grow or form colonies on YMA supplemented with varying concentrations of sodium chloride were characterized. A 20 µl (≈10^9^–10^10^ cells/ml) volume of each bacterial isolate was dropped on a YMA plate containing different concentrations (1%, 2%, 3%, 4%, 5%, 6% and 7%) of NaCl, with 0.01% NaCl as the control^50^.

### Intrinsic antibiotic resistance

Rhizobial growth was tested in YM agar media supplemented with different antibiotics: streptomycin (50, 75, 100, 150, 200, and 500 µg.ml^−1^) and kanamycin (25, 50,75,100, and 150 µg.ml^−1^) with 0 µg.ml^−1^ antibiotic as a control following the procedure of Singh *et al*.^51^. All assays were done in triplicates. The presence or absence of colony growth was evaluated after incubation at 28 °C, and only isolates showing growth in all triplicates plates were considered tolerant, and isolates which did not grow, were considered susceptible to that antibiotic concentration.

### IAA production and phosphate solubilization activities of bacterial isolates

The levels of the root growth-promoting hormone auxin (indole-3-acetic acid) produced by rhizobia in culture filtrate was estimated as described by Raddadi *et al*.^52^. The absorbances of each sample was read at 530 nm and the level of IAA (µg.ml^−1^) calculated using the formula below and information generated by a standard curve of known concentrations (1, 2, 5, 10, 20, 25, 50 and 100 µg/ml IAA) (Supplementary Fig S6):

$${\rm{IAA}}\,(\mu {\rm{g}}/\mathrm{ml})=(y-0.0336)/0.0263$$

where y = sample OD.

The ability of the bacterial isolates to solubilize phosphorus was measured as described by Yanii *et al*.^53^, using tricalcium phosphate. Rhizobial colonies surrounded by a clear zone (halo-zone) were considered to be PSB, and those without, were not PSB.

### Extraction of bacterial genomic DNA and ERIC-PCR amplification

Genomic DNA of the bacterial isolates was extracted from bacterial cells grown in YM broth until the late log phase (10^9^ cell.ml^−1^) using the GenElute bacterial genomic DNA extraction kit (Sigma-Aldrich, USA) according to the manufacturer’s instructions. After DNA extraction, the ERIC (enterobacterial repetitive intergenic consensus) PCR was carried out to amplify the genomic region of isolates using universal primers (Supplementary Table S3). A 25 µl master mix was prepared containing 12.5 µl (2X) myTaq PCR reaction buffer, 1.25 µl each primer pair, 9 µl of nuclease-free PCR water and 1 µl (40–50 ng/µl) of the extracted DNA. The reaction mixture was incubated in a Thermal cycler (T100 Bio-RAD, USA) at standard temperature profiles (Supplementary Table S3). A total volume of 25 µl of the amplified product was mixed with 5 µl loading dye (5x) and then loaded onto 1.2% agarose gel stained with ethidium bromide in a gel electrophoresis system containing 1 X Tris-Acetate EDTA (TAE) and run at 85 V for 270 minutes. Gel imaging and documentation were done using the GEL Doc^TM^ 186 XR+ molecular imager (Bio-RAD, USA).

### ERIC-PCR cluster analysis

Only distinct, well-resolved, unambiguous bands were scored. The lengths of the separated DNA amplified fragments on the gel were calculated using Image Lab software (Bio Rad, USA). The fragments were scored as (1) for the presence of and (0) for the absence of homologous bands. The similarity of the test strains was evaluated using Jaccard’s similarity coefficient, and a dendrogram constructed using Jaccard’s similarity coefficient with NTSYSpc 2.1 software^54^.

### Amplification, sequencing and phylogenetic analysis of 16S rRNA, housekeeping and symbiotic genes

The PCR amplification of the genomic regions 16S-rRNA, housekeeping genes (*atpD*, *glnII*, *gyrB* and *recA*) and symbiotic genes (*nifH* and *nodC*) were done using the same procedure followed for ERIC-PCR amplification with respective primer pairs and standard temperature profiles (Supplementary Table S3). The PCR Cleanup kit (NEB, USA) was used to clean the PCR amplified product. The purified PCR products were sequenced by Macrogen (The Netherlands). BioEdit 7.0.0 software was used to assess the quality of the sequences^55^. The BLASTn program was used to find close aligned related species in the NCBI database. The CLUSTALW program of MEGA 7 was used for pairwise and multiple sequence alignments. Phylogenetic trees were generated from the aligned sequences using the MEGA7 software^56^ with evolutionary distances using the Kimura 2-parameter model, and evolutionary history was inferred using maximum likelihood method algorithm with 1000 bootstraps support^57^. The generated nucleotide sequences of each test gene were submitted to NCBI Genbank.

### Statistical analysis

The influence of soil factors on distribution of bacteria was assessed using canonical correspondence analysis (CCA) with the “vegan” package (version 2.4–2)^58^ of R software^59^. The chemical characteristics of soil such as pH, N, K, P and Ca, and ERIC-PCR genomic fingerprinting data were used for the CCA analysis. The correlation of the canonical axes with the explanatory matrix was determined using the general permutation test. The bi-plot CCA graph was presented for only the soil factors which showed significant effect on bacterial distribution.

All quantitative symbiotic data [nodulation and relative effectiveness, shoot DM and leaf chlorophyll content, as well as photosynthesis (A) and stomatal conductance (gs)] collected from the glasshouse experiments were subjected to test of normal distribution before analysis of variance (ANOVA), using the Statistica data analysis software version 10.0^60^. For each of the datasets, the mean ≈ median, while the kurtosis and skewness values ranged between −0.42 to +0.75 and −0.08 to +1.90, which fall within the range of values (±2) characteristic of normally distributed data^61^. Where significant differences were found, the means were separated using the Duncan’s multiple range test at p ≤ 0.05.

## Supplementary information

Supplementary datasets

## Data Availability

Datasets generated and/or analysed in this study are available from the corresponding author on reasonable request. Nucleotide sequences have been deposited in NCBI GenBank under the accession numbers for 16S rRNA gene (MK611707 - MK611724, MK611726, MK611727, MK611729 - MK611733), *atpD* gene (MK616681 - MK616684, MK616688 - MK616690, MK616692 - MK616700, MK690375 - MK690388), *glnII* gene (MK616701 - MK616736), *gyrB* gene (MK616737 - MK616770), *recA* gene (MK616828 - MK616840, MK616842 - MK616862), *nifH* gene (MK616771 - MK616776, MK616778 - MK616804) and *nodC* gene (MK616805 - MK616827).

## References

[1] Bamshaiye OM, Adegbola JA, Bamishaiye EI (2011). Bambara groundnut: an under-utilized nut in Africa. Adv. Agric. Biotechnol..

[2] Effa, E. B. & Uko, A. E. Food security potentials of Bambara groundnut (*Vigna subterranea* (L.) Verdc.). *Int. J. Dev. Sustain*. **6**, 1919–1930 (2017).

[3] Puozaa, D. K., Jaiswal, S. K. & Dakora, F. D. African origin of *Bradyrhizobium* populations nodulating Bambara groundnut (*Vigna subterranea* L. Verdc) in Ghanaian and South African soils. *PLoS One* **12** (2017). 10.1371/journal.pone.0184943 PMC5612659 28945783

[4] Mazahib AM, Nuha MO, Salawa IS, Babiker EE (2013). Some nutritional attributes of bambara groundnut as influenced by domestic processing. Int. Food Res. J..

[5] Mkandawire FL, Sibuga KP (2002). Yield response of bambara groundnut to plant population and seedbed type. African Crop Sci. J..

[6] Hillocks, R. J., Bennett, C. & Mponda, O. M. Bambara nut: A review of utilisation, market potential and crop improvement. *African Crop Sci*. *J*. **20** (2012).

[7] Mohale KC, Belane AK, Dakora FD (2014). Symbiotic N nutrition, C assimilation, and plant water use efficiency in Bambara groundnut (*Vigna subterranea* L. Verdc) grown in farmers’ fields in South Africa, measured using 15N and 13C natural abundance. Biol. Fertil. soils.

[8] Grönemeyer JL, Kulkarni A, Berkelmann D, Hurek T, Reinhold-Hurek B (2014). Identification and characterization of rhizobia indigenous to the Okavango region in Sub-Saharan Africa. Appl. Environ. Microbiol..

[9] Anand A, Jaiswal SK, Dhar B, Vaishampayan A (2012). Surviving and thriving in terms of symbiotic performance of antibiotic and phage-resistant mutants of *Bradyrhizobium* of soybean (*Glycine max*). Curr. Microbiol..

[10] Msimbira, L. A., Jaiswal, S. K. & Dakora, F. D. Identification and characterization of phages parasitic on bradyrhizobia nodulating groundnut (*Arachis hypogaea* L.) in South Africa. *Appl*. *Soil Ecol*. **108** (2016). 10.1016/j.apsoil.2016.09.010 PMC5176342 28018051

[11] Naamala, J., Jaiswal, S. K. & Dakora, F. D. Antibiotics Resistance in Rhizobium: Type, Process, Mechanism and Benefit for Agriculture. *Curr*. *Microbiol*. **72** (2016). 10.1007/s00284-016-1005-0 26897128

[12] Benson O (2015). Morphological, genetic and symbiotic characterization of root nodule bacteria isolated from Bambara groundnuts (*Vigna subterranea*L. Verdc) from soils of Lake Victoria basin, western Kenya. J. Appl. Biol. Biotechnol..

[13] Mohammed M, Jaiswal S, Dakora F (2018). Distribution and correlation between phylogeny and functional traits of cowpea (*Vigna unguiculata* L. Walp.)-nodulating microsymbionts from Ghana and South Africa. Sci. Rep..

[14] Anyango B, Wilson KJ, Beynon JL, Giller KE (1995). Diversity of Rhizobia Nodulating *Phaseolus vulgaris* L. in Two Kenyan Soils with Contrasting pHs. Appl. Environ. Microbiol..

[15] Prévost D (2003). Cold-adapted rhizobia for nitrogen fixation in temperate regions. Can. J. Bot..

[16] Chemining’wa GN, Vessey JK (2006). The abundance and efficacy of *Rhizobium leguminosarum* bv. viciae in cultivated soils of the eastern Canadian prairie. Soil Biol. Biochem..

[17] Graham, P. H. Ecology of the root-nodule bacteria of legumes. In *Nitrogen-fixing leguminous symbioses* 23–58 (Springer, 2008).

[18] Mothapo NV, Grossman JM, Maul JE, Shi W, Isleib T (2013). Genetic diversity of resident soil rhizobia isolated from nodules of distinct hairy vetch (*Vicia villosa* Roth) genotypes. Appl. soil Ecol..

[19] Puozaa DK, Jaiswal SK, Dakora FD (2019). Phylogeny and distribution of Bradyrhizobium symbionts nodulating cowpea (*Vigna unguiculata* L. Walp) and their association with the physicochemical properties of acidic African soils. Syst. Appl. Microbiol..

[20] Jaiswal, S. K. & Dakora, F. D. Widespread distribution of highly adapted *Bradyrhizobium* species nodulating diverse legumes in Africa. *Front*. *Microbiol*. **10** (2019). 10.3389/fmicb.2019.00310 PMC6395442 30853952

[21] Jaiswal SK, Beyan SM, Dakora FD (2016). Distribution, diversity and population composition of soybean-nodulating bradyrhizobia from different agro-climatic regions in Ethiopia. Biol. Fertil. Soils.

[22] Chidebe IN, Jaiswal SK, Dakora FD (2018). Distribution and phylogeny of microsymbionts associated with cowpea (*Vigna unguiculata*) nodulation in three agroecological regions of Mozambique. Appl. Environ. Microbiol..

[23] Li JH, Wang ET, Chen WF, Chen WX (2008). Genetic diversity and potential for promotion of plant growth detected in nodule endophytic bacteria of soybean grown in Heilongjiang province of China. Soil Biol. Biochem..

[24] Pandya M, Naresh Kumar G, Rajkumar S (2013). Invasion of rhizobial infection thread by non-rhizobia for colonization of Vigna radiata root nodules. FEMS Microbiol. Lett..

[25] Rightmyer AP, Long SR (2011). Pseudonodule formation by wild-type and symbiotic mutant Medicago truncatula in response to auxin transport inhibitors. Mol. plant-microbe Interact..

[26] Hirsch AM (1984). Rhizobium meliloti nodulation genes allow Agrobacterium tumefaciens and Escherichia coli to form pseudonodules on alfalfa. J. Bacteriol..

[27] Laurette NN (2015). Isolation and screening of indigenous bambara groundnut (*Vigna subterranea*) nodulating bacteria for their tolerance to some environmental stresses. Am. J. Microbiol. Res..

[28] Han LL (2009). Unique community structure and biogeography of soybean rhizobia in the saline-alkaline soils of Xinjiang, China. Plant Soil.

[29] Ndungu SM (2018). Cowpea (*Vigna unguiculata* L. Walp) hosts several widespread bradyrhizobial root nodule symbionts across contrasting agro-ecological production areas in Kenya. Agric. Ecosyst. Environ..

[30] Zhang XX (2014). Genetic divergence of *Bradyrhizobium* strains nodulating soybeans as revealed by multilocus sequence analysis of genes inside and outside the symbiosis island. Appl. Environ. Microbiol..

[31] Zhao L (2014). Distribution and diversity of rhizobia associated with wild soybean (*Glycine soja* Sieb. & Zucc.) in Northwest China. Syst. Appl. Microbiol..

[32] Naamala J, Jaiswal SK, Dakora FD (2016). Microsymbiont diversity and phylogeny of native bradyrhizobia associated with soybean (*Glycine max* L. Merr.) nodulation in South African soils. Syst. Appl. Microbiol..

[33] Zinga, M. K., Jaiswal, S. K. & Dakora, F. D. Presence of diverse rhizobial communities responsible for nodulation of common bean (*Phaseolus vulgaris*) in South African and Mozambican soils. *FEMS Microbiol*. *Ecol*. **93** (2017). 10.1093/femsec/fiw236 27915286

[34] Gyogluu C, Mohammed M, Jaiswal SK, Kyei-Boahen S, Dakora FD (2017). Assessing host range, symbiotic effectiveness, and photosynthetic rates induced by native soybean rhizobia isolated from Mozambican and South African soils. Symbiosis.

[35] Gyogluu C, Jaiswal SK, Kyei-Boahen S, Dakora FD (2018). Identification and distribution of microsymbionts associated with soybean nodulation in Mozambican soils. Syst. Appl. Microbiol..

[36] Mohammed M, Jaiswal SK, Dakora FD (2019). Insights into the phylogeny, nodule functioning and biogeographic distribution of microsymbionts nodulating the orphan Kersting’s groundnut [*Macrotyloma geocarpum* (Harms) Marechal & Baudet] in African soils. Appl. Environ. Microbiol..

[37] Chang, Y. L., Wang, J. Y., Wang, E. T. & Liu, H. C. *Bradyrhizobium lablabi* sp. nov., isolated from effective nodules of Lablab purpureus and *Arachis hypogaea*. **234**, 2496–2502 (2011). 10.1099/ijs.0.027110-0 21112989

[38] Ramírez-Bahena MH (2009). *Bradyrhizobium pachyrhizi* sp. nov. and *Bradyrhizobium* jicamae sp. nov., isolated from effective nodules of *Pachyrhizus erosus*. Int. J. Syst. Evol. Microbiol..

[39] Grönemeyer JL, Hurek T, Bünger W, Reinhold-Hurek B (2016). *Bradyrhizobium* vignae sp. nov., a nitrogen-fixing symbiont isolated from effective nodules of *Vigna and Arachis*. Int. J. Syst. Evol. Microbiol..

[40] Bedi MK, Naglot A (2011). Characterization of *Rhizobium* isolated from root nodules of Trifolium alexandrinum. J. Agric. Technol..

[41] Minamisawa K, Fukai K (1991). Production of indole-3-acetic acid by *Bradyrhizobium* japonicum: a correlation with genotype grouping and rhizobitoxine production. Plant cell Physiol..

[42] Appunu C, Sasirekha N, Prabavathy VR, Nair S (2009). A significant proportion of indigenous rhizobia from India associated with soybean (*Glycine max* L.) distinctly belong to *Bradyrhizobium* and *Ensifer* genera. Biol. Fertil. Soils.

[43] Traoré L, Nakatsu CH, DeLeon A, Stott DE (2013). Characterization of six phosphate-dissolving bacteria isolated from rhizospheric soils in Mali. Afr. J. Mocrobiol. Res.

[44] Yulianti, E. & Rakhmawati, A. Screening and characterization of phosphate solubilizing bacteria from isolate of thermophilic bacteria. In *AIP Conference Proceedings* **1868**, 90015 (2017).

[45] Bray RH, Kurtz LT (1945). Determination of total, organic, and available forms of phosphorus in soils. Soil Sci..

[46] Somasegaran, P. & Hoben, H. Handbook for *Rhizobia Methods* in Legume-*Rhizobium* Technology. (Springer-Verlag, 1994).

[47] Broughton WJ, Dilworth MJ (1971). Control of leghaemoglobin synthesis in snake beans. Biochem. J..

[48] Richardson AD, Duigan SP, Berlyn GP (2002). An evaluation of noninvasive methods to estimate foliar chlorophyll content. New Phytol..

[49] Chibeba AM, Kyei-Boahen S, de Fátima Guimarães M, Nogueira MA, Hungria M (2018). Feasibility of transference of inoculation-related technologies: A case study of evaluation of soybean rhizobial strains under the agro-climatic conditions of Brazil and Mozambique. Agric. Ecosyst. Environ..

[50] Moschetti G (2005). Use of nodulation pattern, stress tolerance, nodC gene amplification, RAPD-PCR and RFLP-16S rDNA analysis to discriminate genotypes of *Rhizobium leguminosarum* biovar viciae. Syst. Appl. Microbiol..

[51] Singh SK, Jaiswal SK, Vaishampayan A, Dhar B (2013). Physiological behavior and antibiotic response of soybean (*Glycine max* L.) nodulating rhizobia isolated from Indian soils. African. J. Microbiol. Res..

[52] Raddadi N, Cherif A, Boudabous A, Daffonchio D (2008). Screening of plant growth promoting traits of Bacillus thuringiensis. Ann. Microbiol..

[53] Yanni YG (2001). The beneficial plant growth-promoting association of *Rhizobium* leguminosarum bv. trifolii with rice roots. Funct. Plant Biol..

[54] Rohlf, F. J. Applied Biostatistics, I. & Exeter Software (Firm). *NTSYS-pc*: *numerical taxonomy and multivariate analysis system*. (Applied Biostatistics, Inc., 2009).

[55] Hall, T. BioEdit version 7.0. 0. Distributed by the author, website, www.mbio.ncsu.edu/BioEdit/bioedit.html (2004).

[56] Kumar, S., Stecher, G. & Tamura, K. MEGA7: Molecular Evolutionary Genetics Analysis Version 7. 0 for Bigger Datasets. **33**, 1870–1874 (2016). 10.1093/molbev/msw054 PMC8210823 27004904

[57] Felsenstein J (1985). Confidence limits on phylogenies: an approach using the bootstrap. Evolution (N. Y)..

[58] Oksanen, J. *et al*. vegan: community ecology package, version 2.4-1, R Foundation for Statistical Computing (2016).

[59] R Core Team. R: A language and environment for statistical computing, R foundation for Statistical Computing, Vienna, Austria (2015).

[60] Statsoft Inc. Statistica (data analysis software system). version 10, www.statsoft.com (2011).

[61] Holmes, D., Moody, P., Dine, D. & Trueman, L. *Research methods for the biosciences*. (Oxford university press, 2017).

