# Supplementary material for: Symbiotic effectiveness and ecologically adaptive traits of native rhizobial symbionts of Bambara groundnut (Vigna subterranea L. Verdc.) in Africa and their relationship with phylogeny
Source: Sci Rep. 2019 Sep 2;9:12666. doi: 10.1038/s41598-019-48944-1 (PMC6718677; doi:10.1038/s41598-019-48944-1)
Supplement: Supplementary file 1 — Supplementary datasets [file 41598_2019_48944_MOESM1_ESM.pdf]

Symbiotic effectiveness and ecologically adaptive traits of native rhizobial symbionts of Bambara groundnut (*Vigna subterranea* L. Verdc.) in Africa and their relationship with phylogeny

Fadimata Y. I. Ibny, Sanjay K. Jaiswal, Mustapha Mohammed, Felix D. Dakora

Table S1: Information of nucleotide sequences used in the phylogeny analysis

Locus	Number of strains used for tree construction	Number of conserved sites	Number of variable sites	Number of parsimony-informative sites	Number of singleton sites	Total	T/C/A/G
16S rRNA	69	473 (77.67)	132 (21.67)	28 (4.60)	104 (17.07)	609	19.8/21.8/26.1/32.4
atpD	66	147 (60.74)	95 (39.26)	63 (26.03)	41 (13.22)	242	19.8/33.3/16.8/30.1
glnII	76	264 (50.87)	255 (49.13)	162 (31.21)	93 (17.92)	519	16.4/32.6/20.1/30.8
gyrB	71	266 (50.76)	258 (49.24)	162 (30.92)	96 (18.32)	524	16.3/32.8/20.0/30.9
nifH	67	164 (59.85)	110 (40.15)	95(34.67)	15 (5.47)	274	20.2/26.3/19.9/33.5
nodC	35	36 (16.36)	184 (83.64)	167 (75.91)	17 (7.73)	220	21.5/29.5/21.1/27.9
recA	76	123 (58.29)	88 (41.71)	58 (27.49)	30 (14.22)	211	16.9/30.3/14.8/38.0
Concatenated *(atpD+gln+gyr+recA)*	60	827(57.91)	601(42.1)	397(27.8)	204(14.29)	1428	17.1/32.5/18.6/31.7

Table S2 Nodulation, plant growth, gas exchange parameters and relative effectiveness (RE) elicited by native rhizobial symbionts of Bambara groundnut. Values (Mean ± S.E) followed by dissimilar letters in a column are significantly different at ***p<0.001.

Isolate	Nodule number plant^{-1}	Nodule DM mg.plant^{-1}	Shoot DM g.plant^{-1}	Total Chl (mg. g^{-1}. Fresh wt.)	A µmol (CO_2) ms^{-2}s^{-1}	gs mol (H_2O) m^{-2}s^{-1}	RE %
TUTMa1	84±3.2q-x	182±10.4s-y	2.87±0.06s-A	1.16±0.08E-K	14.9±0.32o-y	0.26±0.02l-y	107±2.4w-A
TUTMa2	108±2.9j-n	211±12.4o-t	3.05±0.05p-x	1.71±0.05h-m	14.6±1.18q-y	0.28±0.05l-x	114±2.0q-x
TUTMa3	57±0.3C-K	257±33.7h-n	3.53±0.27h-n	1.71±0.05h-m	14.2±0.35r-z	0.29±0.01l-w	132±10.1h-o
TUTMa4	61±4.6A-J	183±17.3s-y	2.24±0.07DEF	1.33±0.01x-F	16.0±0.05j-s	0.31±0.00j-u	83±2.4EFG
TUTMa5	73±9.0v-G	278±12.4f-j	3.24±0.08l-s	0.96±0.12K-O	15.4±1.19m-w	0.32±0.04h-u	121±3.0n-u
TUTMa6	101±2.9l-q	347±15.4abc	4.19±0.08fg	1.17±0.01D-K	15.1±0.41n-x	0.36±0.03f-p	156±3.0fg
TUTMa7	111±4.9i-m	234±10.4k-q	3.00±0.16q-y	1.50±0.07m-y	15.2±0.20n-x	0.37±0.01f-p	112±6.0r-y
TUTMa8	116±0.9i-l	251±4.6i-n	3.88±0.01gh	2.23±0.06bc	12.5±0.11z-D	0.23±0.02r-B	145±0.4gh
TUTMa9	104±2.3k-p	243±16.5j-o	3.12±0.12o-v	1.26±0.05z-H	17.7±0.15e-k	0.59±0.01a	116±4.6q-w
TUTMa10	84±6.6q-x	198±8.7q-w	3.08±0.03p-w	1.68±0.16h-p	10.2±0.53EFG	0.14±0.01A-D	115±1.2q-x
TUTMa11	147±2.9fg	362±15.2a	3.70±0.15hij	1.51±0.06k-y	15.0±0.74o-y	0.26±0.03m-y	138±5.5h-k
TUTMa12	110±1.7j-m	312±1.7c-f	3.77±0.00hi	1.64±0.07i-r	18.0±0.04e-i	0.35±0.00f-q	141±0.2h-i
TUTMa13	77±9.3r-B	338±17.3abc	3.40±0.07j-p	1.12±0.03F-L	16.4±1.07h-q	0.38±0.07e-n	127±2.5j-q
TUTMa14	71±0.0w-H	272±17.9g-k	3.01±0.07q-y	1.47±0.07q-z	15.6±0.80l-u	0.47±0.02b-f	112±2.7r-y
TUTMa15	145±11.3fgh	347±15.6abc	3.61±0.01h-l	1.59±0.04j-u	13.8±0.64u-A	0.24±0.05q-A	135±0.5h-m
TUTMa16	140±2.6gh	342±10.5abc	3.76±0.09hi	1.51±0.00k-y	14.7±0.11p-y	0.29±0.01k-v	140±3.5hi
TUTMa17	90±4.0n-v	218±9.2n-s	3.26±0.07l-r	1.69±0.05h-o	14.9±0.08o-y	0.29±0.02l-v	122±2.6m-s
TUTMa18	171±10.7e	207±1.3o-u	4.23±0.06f	1.71±0.07h-m	17.7±0.62e-k	0.42±0.10b-j	158±2.4f
TUTMa19	62±6.4z-J	279±6.6f-j	3.69±0.06h-k	1.15±0.11E-K	18.5±0.34c-g	0.44±0.08b-h	138±2.3h-l
TUTMa20	267±11.0c	320±6.9b-e	5.80±0.13b	1.62±0.03j-s	18.1±0.29e-i	0.46±0.00b-f	216±4.7b
TUTMa21	120±4.3ijk	272±15.2g-k	6.18±0.08a	1.71±0.04h-l	19.0±0.47b-f	0.44±0.03b-i	231±3.1a
TUTMa22	128±4.0h-i	316±18.8cde	3.26±0.24l-r	1.49±0.01n-y	19.5±1.09b-e	0.50±0.04abc	121±9.0m-t
TUTMa23	101±2.6l-q	154±13.6x-E	1.40±0.02K-N	1.66±0.00h-r	16.5±0.78g-q	0.32±0.04j-u	52±0.8L-O
TUTMa24	110±2.6j-m	230±4.6l-r	2.94±0.05r-z	1.66±0.00h-r	17.9±1.36e-j	0.45±0.05b-g	110±1.8s-z
TUTMa25	58±2.9C-K	175±11.3t-A	1.70±0.18IJK	1.53±0.02j-x	18.3±1.22c-h	0.36±0.02f-q	63±6.8JKL
TUTMa26	96±1.7m-r	230±16.2l-r	2.30±0.10C-F	1.95±0.09def	14.8±0.15p-y	0.29±0.04k-v	86±3.9C-G
TUTMa27	74±2.0u-E	169±0.9u-B	2.26±0.18DEF	1.72±0.01h-k	15.7±0.42l-u	0.34±0.02g-s	84±6.9D-G
TUTMa28	55±2.0E-K	121±3.8E-K	1.29±0.05L-O	1.63±0.01i-s	15.2±0.02n-x	0.46±0.00b-g	48±1.9MNO
TUTMa29	65±8.4y-I	175±5.2t-A	2.81±0.13u-A	0.94±0.01L-O	17.0±0.05g-n	0.32±0.05h-u	105±4.7w-A
TUTMa30	26±1.2KL	117±8.2E-K	1.28±0.03L-O	1.34±0.03w-E	12.5±0.56z-D	0.17±0.02w-D	48±1.3MNO
TUTMa31	106±0.3j-o	110±3.9G-K	2.59±0.13z-D	1.66±0.19h-r	16.4±0.50h-q	0.35±0.02f-r	97±4.8z-D
TUTMa32	60±4.9B-K	150±11.3y-F	1.13±0.00M-P	1.23±0.03A-H	17.6±0.22e-l	0.32±0.03i-u	42±0.2NP
TUTMa33	46±3.8IJK	93±7.2JK	0.85±0.07PQ	0.91±0.01MNO	16.3±0.05i-q	0.22±0.00t-C	32±2.7PQ
TUTMa34	58±0.9C-K	93±3.8JK	1.15±0.00M-P	1.48±0.01p-y	13.8±0.67t-A	0.32±0.00h-u	43±0.1NOP
TUTMa35	92±3.5m-u	172±20.2JK	1.31±0.08L-O	0.98±0.03K-O	15.2±0.04n-x	0.22±0.00s-C	49±2.8MNO
TUTMa36	56±6.1D-K	99±16.5H-K	0.73±0.04Q	0.84±0.06O	13.3±0.10x-B	0.35±0.04f-q	27±1.6Q
TUTMa37	84±2.0q-x	191±6.9r-x	2.29±0.04C-F	1.31±0.05y-G	13.9±1.40t-A	0.37±0.11f-o	86±1.3C-G
TUTMa38	151±5.2fg	261±10.1h-l	2.59±0.19z-D	1.23±0.06A-H	15.9±0.05j-s	0.49±0.06a-e	97±6.9z-D
TUTMa39	93±0.3m-t	155±6.1x-E	2.17±0.02EFG	1.22±0.03A-H	12.0±0.58A-E	0.25±0.01o-A	81±0.9FGH
TUTMa40	25±1.7L	88±0.0K	1.07±0.05N-P	1.01±0.04I-O	10.0±0.64FG	0.17±0.00x-D	40±1.9OP
TUTMa41	59±4.2B-K	223±11.8l-r	2.73±0.15w-B	1.51±0.10k-y	15.2±0.57n-x	0.38±0.01d-m	102±5.7z-B
TUTMa42	73±6.4v-F	200±14.4q-w	2.10±0.06E-H	1.49±0.11n-y	13.1±0.09y-C	0.23±0.03q-A	78±2.1FGH
TUTMa43	63±2.6y-I	166±5.8v-C	2.64±0.14y-C	1.23±0.05A-H	15.6±0.04l-u	0.31±0.04j-v	98±5.3z-C
TUTMa44	76±4.9s-C	286±2.0e-i	3.49±0.19i-o	1.33±0.04w-E	11.0±0.11D-G	0.19±0.02v-D	130±7.1i-p
TUTMa45	78±0.9r-B	126±12.0D-K	1.31±0.02L-O	1.53±0.07j-x	16.0±0.14j-s	0.37±0.01f-o	49±0.6MNO
TUTMa46	74±1.7t-D	221±27.3m-s	2.75±0.11v-B	1.20±0.05B-I	15.9±0.75k-s	0.37±0.01f-o	103±4.3z-B
TUTMa47	116±4.0i-l	210±8.1o-t	3.19±0.06m-t	1.05±0.00H-N	16.7±0.12g-p	0.36±0.00f-q	119±2.1o-v
TUTMa48	18±0.9L	118±11.0E-K	1.53±0.06JKL	1.40±0.07t-C	14.9±1.00o-y	0.26±0.06n-z	57±2.3KLM
TUTMa49	52±5.2H-K	126±11.2D-K	1.46±0.08KLM	1.42±0.02s-A	15.1±0.49n-x	0.38±0.01e-n	54±2.9LMN
TUTMa50	44±4.3JK	134±8.9B-I	1.55±0.06JKL	1.38±0.04u-C	11.4±0.03C-G	0.22±0.00s-C	58±2.1KLM
TUTMa51	82±2.6r-y	261±15.3h-l	2.75±0.05v-B	1.72±0.05h-k	16.2±0.40i-r	0.34±0.02g-t	103±1.9x-B
TUTMa52	60±1.2B-K	195±6.1q-w	2.90±0.11r-A	1.74±0.02g-j	15.5±0.64m-v	0.33±0.06h-t	108±4.2t-A
TUTMa53	41±0.7KL	142±9.5z-G	1.40±0.09K-N	1.42±0.09s-A	9.8±0.00G	0.11±0.00CDE	52±3.3L-O
TUTMa54	86±6.9p-x	168±6.6u-B	2.04±0.11F-H	1.38±0.00v-C	15.7±0.44l-u	0.28±0.02l-y	76±3.9GHI
TUTMa55	105±5.8j-o	163±5.1v-D	1.98±0.08F-H	0.87±0.01NO	14.7±0.60p-y	0.16±0.00y-D	74±2.8G-J
TUTMa56	160±9.0ef	332±4.6a-d	4.11±0.02fg	1.38±0.01v-D	17.3±0.14f-m	0.39±0.07c-l	153±0.9fg
TUTMa57	142±3.8gh	234±5.5k-q	1.39±0.05K-N	1.01±0.02I-O	20.0±0.04bcd	0.36±0.00f-q	52±1.8L-O
TUTMa58	42±1.7K	177±11.3t-z	2.13±0.04EFG	1.70±0.01h-n	20.6±0.69ab	0.50±0.03a-d	79±1.3FGH
TUTMa59	115±4.6i-l	226±0.3l-r	2.89±0.05r-A	1.56±0.02j-v	14.1±0.61s-z	0.28±0.02l-x	108±1.8u-A
TUTMa60	71±0.9w-H	228±17.2l-r	2.55±0.09A-D	1.60±0.04j-t	22.1±0.65a	0.53±0.02ab	95±3.2A-E
TUTMa61	64±7.5y-I	160±19.6w-D	1.54±0.03JKL	1.35±0.03v-E	16.9±0.45g-o	0.45±0.05b-g	57±1.3KLM
TUTNa62	295±2.9b	263±3.8g-l	4.38±0.08ef	1.45±0.14r-z	18.2±0.34c-i	0.42±0.03c-k	163±3.1ef
TUTNa63	54±5.5G-K	170±11.8u-B	2.60±0.02z-D	1.33±0.03x-F	14.1±0.86s-z	0.31±0.04j-v	97±0.9z-D
TUTNou64	81±2.0r-z	175±0.6t-A	3.15±0.04o-u	1.40±0.07t-B	20.0±0.30bc	0.36±0.00f-p	118±1.6p-w
TUTNou65	54±1.7F-K	136±1.6A-H	1.75±0.14H-K	1.23±0.00A-H	13.4±0.02w-B	0.25±0.00o-A	65±5.3I-L
TUTNou66	88±0.9o-w	278±12.3f-j	3.32±0.05k-q	2.08±0.04cd	17.3±1.27f-m	0.33±0.08h-t	124±1.9m-r
TUTNou67	296±20.5b	291±10.7e-h	3.34±0.18j-q	1.40±0.03t-C	13.6±0.04v-B	0.27±0.01l-y	125±6.6l-r
TUTNou68	218±10.1d	320±0.9b-e	4.87±0.04cd	1.55±0.08j-w	15.8±0.39k-t	0.29±0.04l-x	182±1.6cd
TUTNou69	74±9.2t-D	166±18.8v-c	2.69±0.09x-B	1.38±0.02v-D	16.0±0.01j-s	0.36±0.00f-q	100±3.3y-B
TUTNou70	91±2.0n-v	256±6.6h-n	2.86±0.06t-A	1.16±0.01E-K	16.4±0.38h-q	0.26±0.01m-y	107±2.3v-A
TUTNou71	60±5.2B-K	129±11.8C-J	1.70±0.01IJK	1.21±0.01A-I	11.0±0.20D-G	0.12±0.01B-E	63±0.4JKL
TUTNou72	44±0.3JK	112±6.1F-K	1.84±0.00G-J	0.83±0.01O	10.7±0.69D-G	0.20±0.02u-C	69±0.1H-K
TUTNou73	94±0.9m-s	176±13.1t-z	2.43±0.13B-E	0.98±0.05J-O	17.6±0.52f-l	0.28±0.01l-y	91±5.0B-F
TUTNou74	57±5.0C-K	104±1.8G-K	1.69±0.15IJK	1.11±0.01G-L	11.8±0.46B-F	0.14±0.01z-D	63±5.7JKL
TUTNou75	287±4.0b	300±6.1d-g	3.56±0.13h-m	1.48±0.04o-y	15.4±0.21m-w	0.27±0.01l-y	133±4.8h-m
TUTNou76	80±4.3r-A	203±5.6o-v	3.17±0.02n-u	1.26±0.06z-H	17.3±0.01f-m	0.30±0.00j-v	118±0.9o-w
TUTka77	228±9.6d	355±10.9ab	6.08±0.01ab	1.83±0.01f-i	ND	ND	227±0.5ab
TUTka78	219±6.1d	276±6.9f-j	4.25±0.13f	2.40±0.06ab	ND	ND	159±4.8f

TUTka79	10±0.3L	96±2.0IJK	1.60±0.14JKL	1.19±0.00C-J	ND	ND	60±5.4KLM
TUTka80	366±11.0a	342±16.7abc	5.01±0.01c	2.24±0.06bc	ND	ND	187±0.2c
TUTka81	88±1.5o-w	117±7.5E-K	2.54±0.02A-D	1.92±0.00d-g	ND	ND	95±0.7A-E
TUTka82	155±6.1efg	259±4.6h-m	4.64±0.23de	2.48±0.06a	ND	ND	173±8.6de
TUTGh83	24±0.6L	52±0.3L	0.83±0.03PQ	1.46±0.00r-z	ND	ND	31±1.2PQ
TUTGh84	151±10.1fg	198±9.9q-w	2.05±0.05F-I	1.86±0.15e-h	ND	ND	76±1.9GHI
TUTGh85	123±11.8ij	240±15.6j-p	3.36±0.25j-q	1.68±0.04h-q	ND	ND	125±9.5j-r
TUTGh87	68±1.9x-J	169±18.5u-B	3.35±0.20j-q	1.64±0.02i-r	ND	ND	125±7.4k-r
TUTGh88	62±0.3z-J	104±1.0G-K	1.56±0.07JKL	1.70±0.18h-n	ND	ND	58±2.6KLM
TUTGh89	141±4.3gh	234±1.2k-q	3.71±0.26hij	2.03±0.10de	ND	ND	138±9.8hij
TUTGh90	106±1.7j-o	203±3.2p-v	2.60±0.03z-D	1.50±0.01l-y	ND	ND	97±1.1z-D
B. CB756	70±3.8w-H	122±11.3E-K	1.87±0.12G-J	1.08±0.01H-M	12.4±0.01z-D	0.24±0.00p-A	70±4.4H-K
5 mM KNO_3	NA	NA	2.68±0.31y-B	0.58±0.03P	7.8±0.64H	0.09±0.01DE	---
Uninoculated	NA	NA	1.00±0.11OPQ	0.23±0.00Q	1.57±0.17I	0.03±0.00E	NA
F statistics	*137.9****	*43.2****	*108.9****	*36.7****	*28.8****	*9.5****	*118.1****

NA=not applicable; ND= not determined

Table S3: Primers and thermal cycling conditions used in the different PCR amplification

Primers	Sequences 5' – 3'	Temperature profiles	References
ERIC F<br>ERIC R	5'ATGTAAGCTCCTGGGGATTCAC 3′<br>5'AAGTAAGTGACTGGGGTGAGCG-3′	95°C for 7 min was followed by 30 cycles at 94°C for 1 min, at 52°C for 1 min, and at 65°C for 8 min; 1 cycle at 65°C for 16 min; and a final soak at 4°C.	1
16S rRNA F<br>16S rRNA R	9-5'AGAGTTTGATCCTGGCTCAG3'-29<br>1474-5' CTTAAGGAGGTGATCCAGCC3'-1494	5 min at 95°C, 35 × (1 min at 95°C, 1 min at 55°C, 1 min at 72°C), 10 min at 72°C	2
*nif*H F<br>*nif*H R	28-3'TACGGNAARGGSGGNATCGGCAA3'-50<br>808-5'AGCATGTCYTCSAGYTCNTCCA3'-787	5 min at 94°C, 20 × [30s at 94°C, 30s at 65°C (-0.5°C\cycle), 90s at 72°C], 25 x (30s at 94°C, 30s at 55°C, 90s at 72°C), 10 min at 72°C	3
*gln*ll F<br>*gln*II R	13-5'AAGCTCGAGTACATCTGGCTCGACGG3'-38<br>681-5'SGAGCCGTTCCAGTCGGTGGTGTCG3'-660	2 min at 95°C, 35 × (45s at 95°C, 30s at 65°C, 90s at 72°C), 10 min at 72°C	4
*gyr*B F<br>*gyr*B R	343-5'TTCGACCAGAAYTCCTAYAAGG3'-364<br>1043-5'AGCTTGTCCTTSGTCTGCG3'-1025	10 min at 95°C, 35 × (30s at 94°C, 30s at 58°C, 1 min at 72°C), 10 min at 72°C	5
*atp*D F<br>*atp*D R	189-5'TCTGGTCCGYGGCCAGGAAG3'-208<br>804-5'CGACACTTCCGARCCSGCCTG3'-784	2 min at 95ºC, 35 × (45s at 95ºC, 30s at 65ºC, 1.5min at 72ºC), 10 min at 72ºC	6
*nod*C F<br>*nod*C R	5'GTC GAT TGC MRG TCA AGA CTA CG3'<br>5'GCC AGG TCT IGT TGC GAT TGC TC3'	30s at 94°C, 40 × (30s at 94°C, 1 min at 55.4°C, 30s at 72°C), 5 min 72°C	7
*rec*A F<br>*rec*A R	CAACTGCMYTGCGTATCGTCGAAGG<br>CGGATCTGGTTGATGAAGATCACCATG	5 min at 95 ºC, 34 × (45s at 98 ºC, 30s at 79.3 ºC, 90s at 72 ºC), 10 min at 72 ºC	4

1. De Bruijn, F. J. Use of repetitive (repetitive extragenic palindromic and enterobacterial repetitive intergeneric consensus) sequences and the polymerase chain reaction to fingerprint the genomes of *Rhizobium meliloti* isolates and other soil bacteria. Appl. Environ. Microbiol. **58(7),** 2180-2187 (1992).
2. Weisburg, W. G., Barns, S. M., Pelletier DA *et al.* 16S ribossomal DNA amplification for phylogenetic study. J. Bacteriol. **173**, 697–703 (1991).

3. Nzoué, A., Miché, L., Klonowska, A., Laguerre, G., de Lajudie, P., & Moulin, L. Multilocus sequence analysis of bradyrhizobia isolated from *Aeschynomene* species in Senegal. Syst. Appl. Microbiol. **32**, 400–412 (2009).
4. Stepkowski, T., Zak, M., Moulin, L., Króliczak, J., Golińska, B., Narozna, D., Safronova, V. I. & Madrzak, C. J. *Bradyrhizobium canariense* and *Bradyrhizobium japonicum* are the two dominant rhizobium species in root nodules of lupin and serradella plants growing in Europe. Syst. Appl. Microbiol. **34**, 368–375 (2011).
5. Marek-Kozaczuk, M., Leszcz, A., Wielbo, J., Wdowiak-Wróbel, S. & Skorupska, A. *Rhizobium pisi* sv. trifolii K3.22 harboring nod genes of the *Rhizobium leguminosarum* sv. trifolii cluster. Syst. Appl. Microbiol. **36**, 252–258 (2013).
6. Jaiswal, S. K., Msimbira, L. A., & Dakora, F. D. Phylogenetically diverse group of native bacterial symbionts isolated from root nodules of groundnut (*Arachis hypogaea* L.) in South Africa. Syst. Appl. Microbiol. **40(4)**, 215-26 (2017).
7. Sterner, J. P. & Parker, M. A. Diversity and Relationships of Bradyrhizobia from *Amphicarpaea bracteata* Based on partial nod and Ribosomal Sequences. Syst. Appl. Microbiol. **22**, 387-392 (1999).

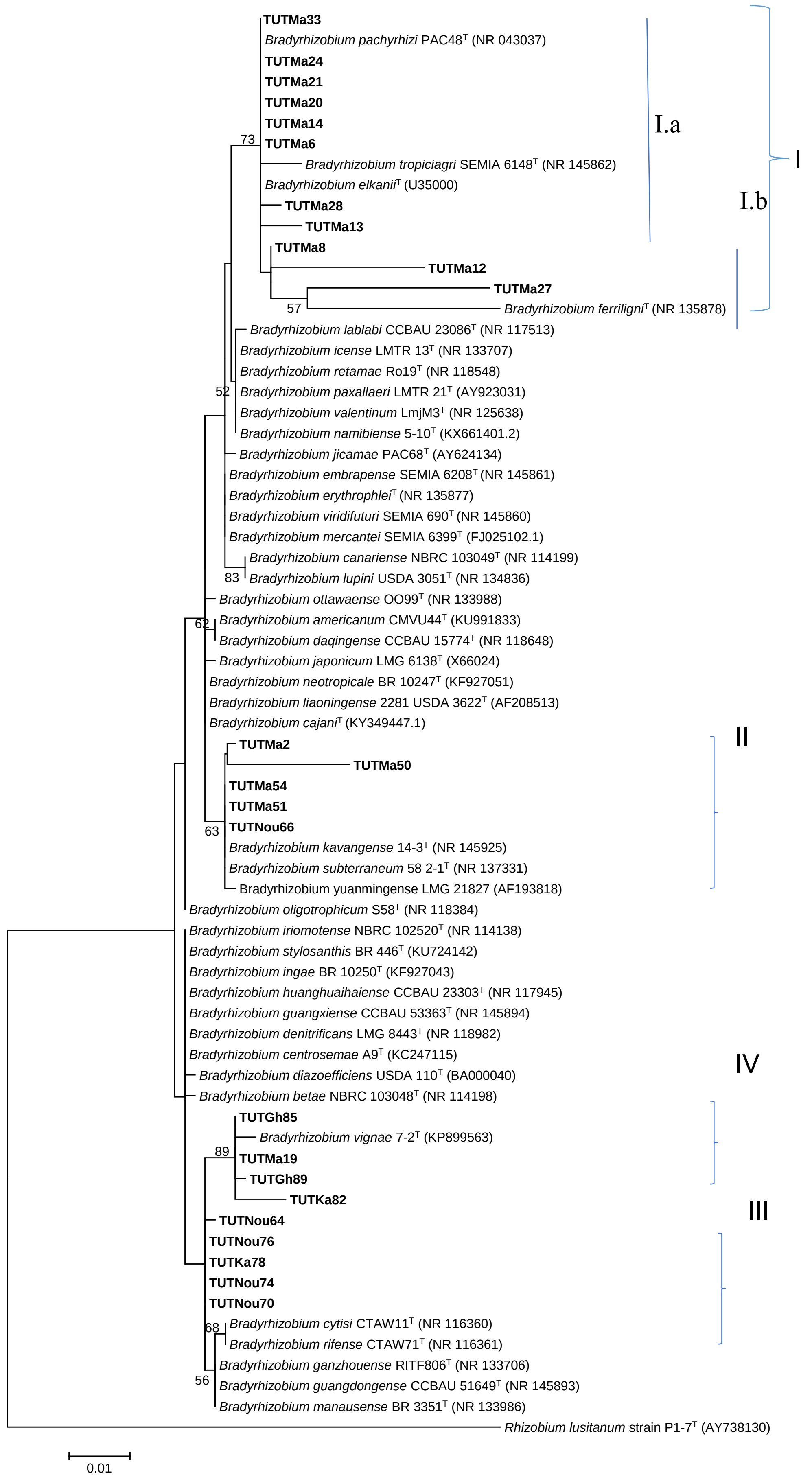

Fig S1: The Maximum-likelihood method based 16S-rRNA phylogeny. Bootstrap values (1000 replicates) are indicated above the branches which shows the percentage of trees in which the associated taxa clustered together.

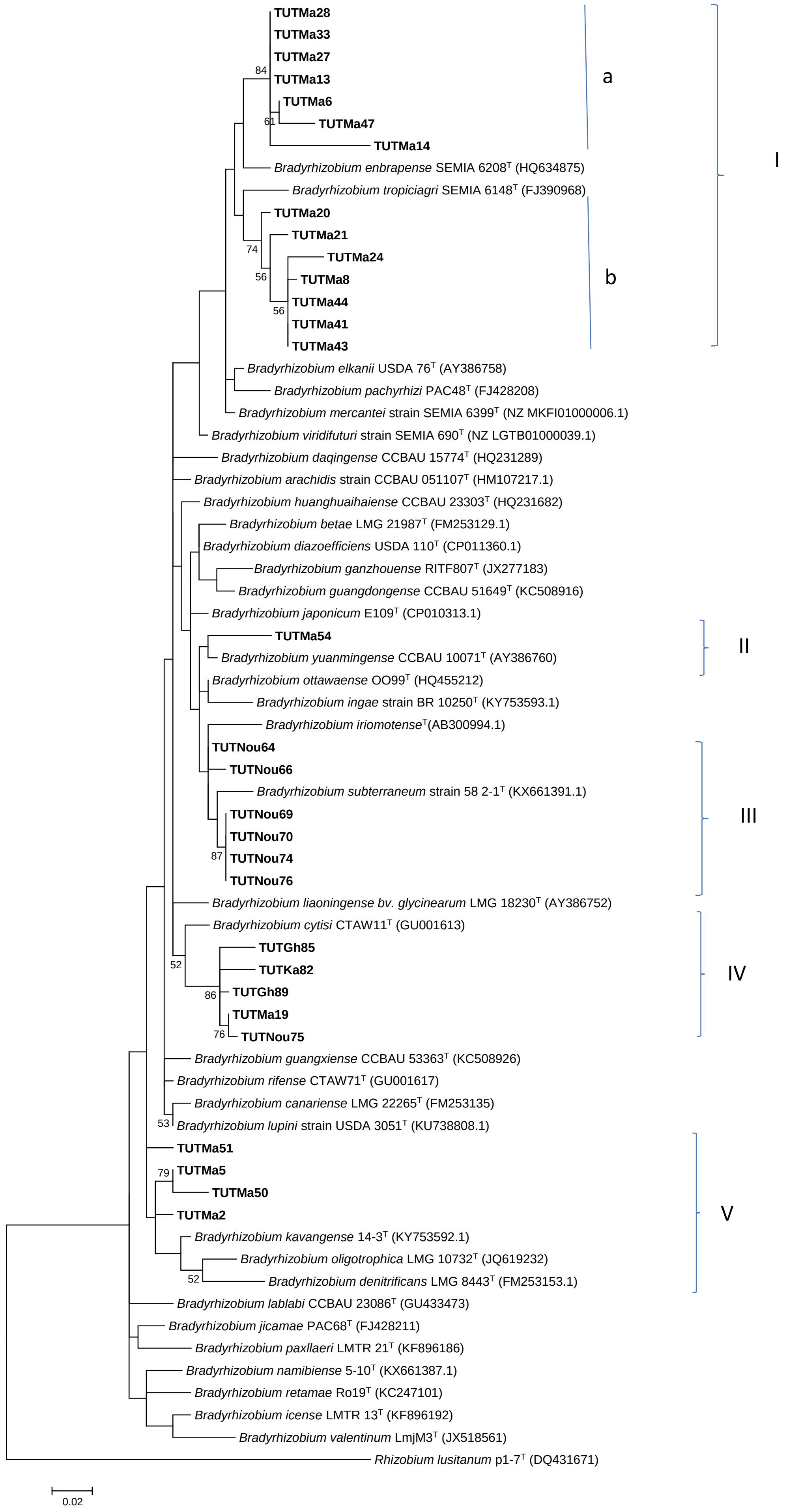

Fig S2: Phylogenetic tree based on atp*D* gene sequences generated by the maximum likelihood method. Bootstrap values (1000 replicates) are indicated above the branches.

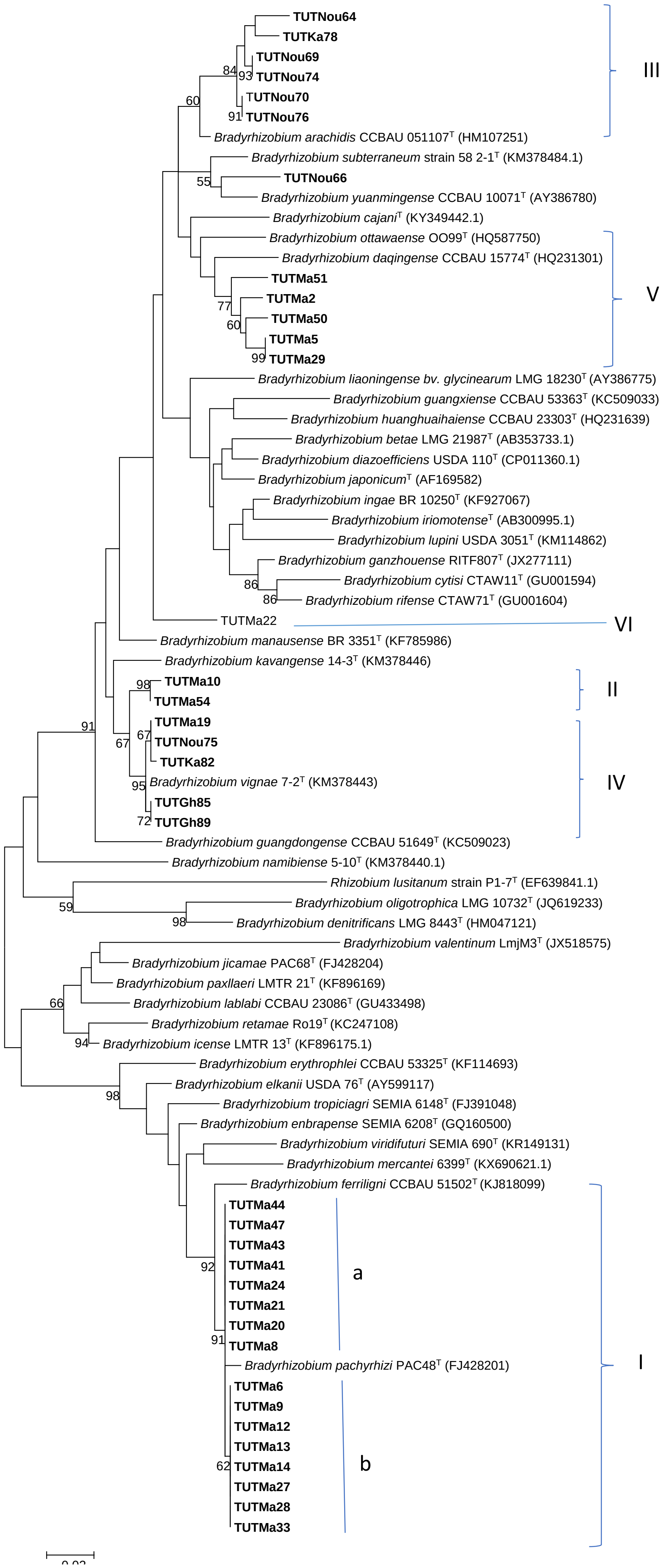

Fig S3: Phylogenetic tree based on *gln*II gene sequences generated by the Maximum likelihood method. Bootstrap values (1000 replicates) are indicated above the branches.

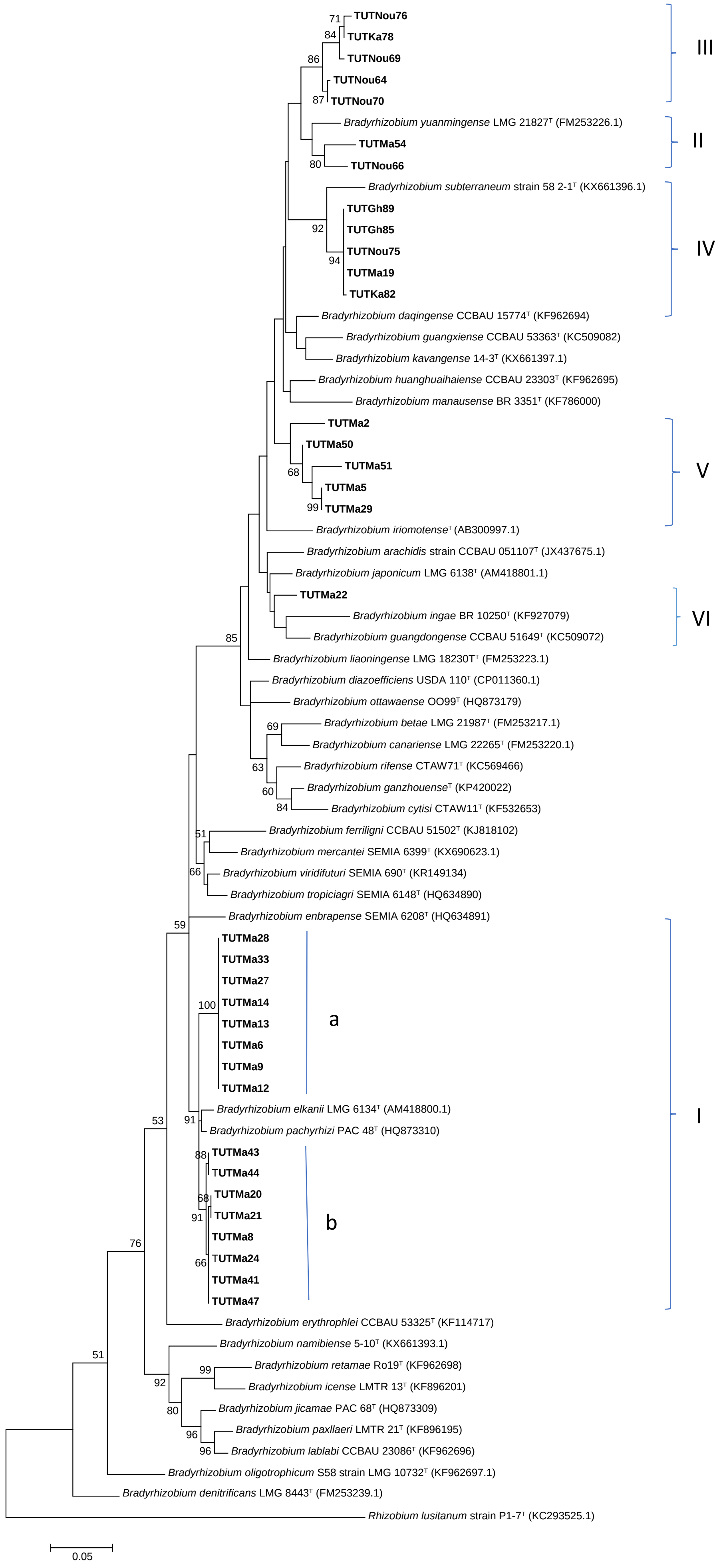

Fig S4: Phylogenetic tree based on *gyr*B gene sequences generated by Maximum likelihood method. Bootstrap values (1000 replicates) are indicated above the branches.

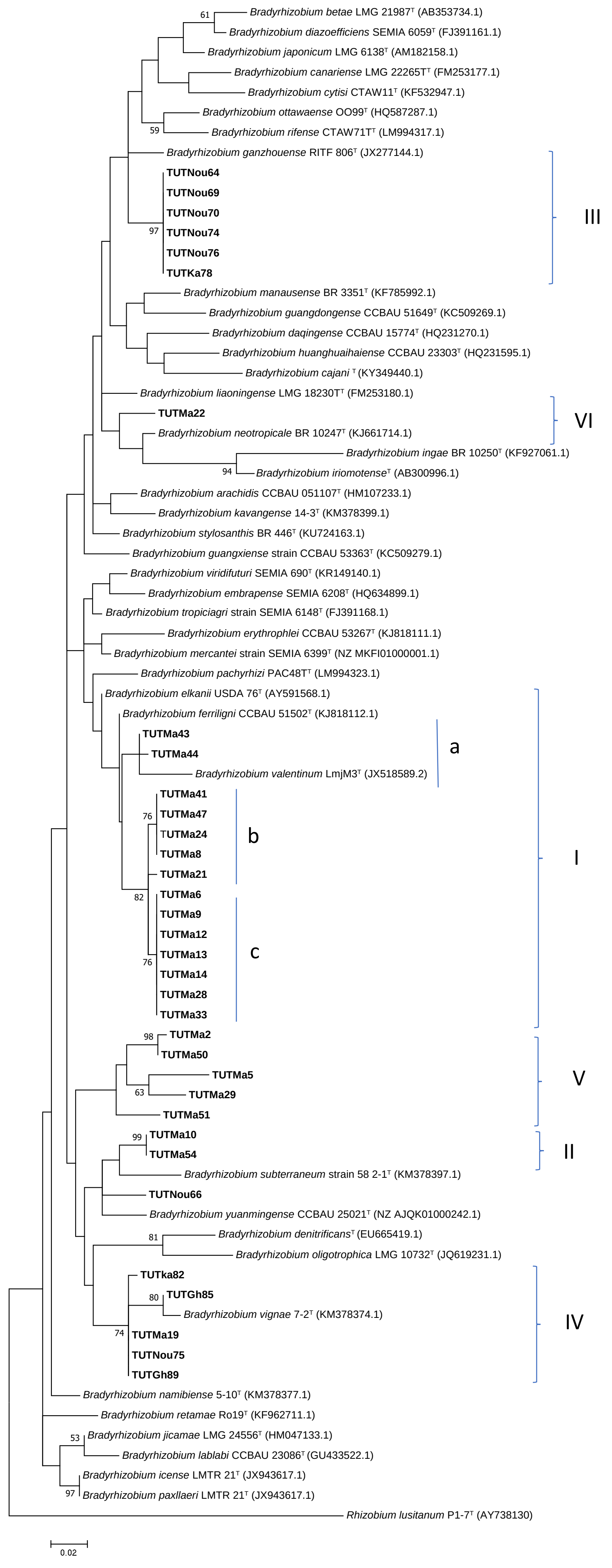

Fig S5: Phylogenetic tree based on *rec*A gene sequences generated by Maximum likelihood method. Bootstrap values (1000 replicates) are indicated above the branches.

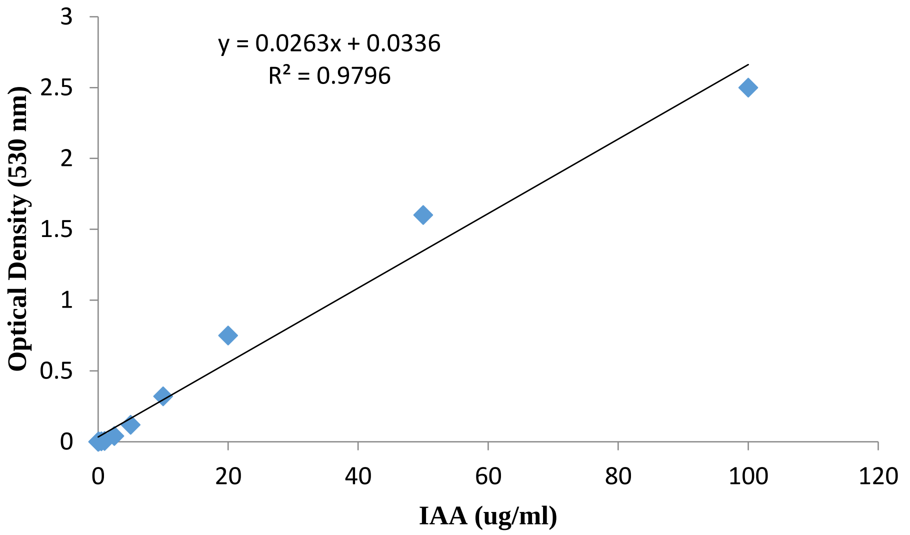

Fig. S6: Standard curve plotted based on optical density data (at 530 nm) of known IAA concentrations
